# *In silico* comprehensive analysis of coding and non-coding SNPs in human mTOR protein

**DOI:** 10.1371/journal.pone.0270919

**Published:** 2022-07-05

**Authors:** Tahirah Yasmin

**Affiliations:** Department of Biochemistry and Molecular Biology, University of Dhaka, Dhaka, Bangladesh; CSIR-IHBT: Institute of Himalayan Bioresource Technology CSIR, INDIA

## Abstract

The mammalian/mechanistic target of rapamycin (mTOR) protein is an important growth regulator and has been linked with multiple diseases including cancer and diabetes. Non-synonymous mutations of this gene have already been found in patients with renal clear cell carcinoma, melanoma, and acute lymphoid leukemia among many others. Such mutations can potentially affect a protein’s structure and hence its functions. In this study, therefore, the most deleterious SNPs of mTOR protein have been determined to identify potential biomarkers for various disease treatments. The aim is to generate a structured dataset of the *mTOR* gene’s SNPs that may prove to be an asset for the identification and treatment of multiple diseases associated with the target gene. Both sequence and structure-based approaches were adopted and a wide variety of bioinformatics tools were applied to analyze the SNPs of mTOR protein. In total 11 nsSNPs have been filtered out of 2178 nsSNPs along with two non-coding variations. All of the nsSNPs were found to destabilize the protein structure and disrupt its function. While R619C, A1513D, and T1977R mutations were shown to alter C alpha distances and bond angles of the mTOR protein, L509Q, R619C and N2043S were predicted to disrupt the mTOR protein’s interaction with NBS1 protein and FKBP1A/rapamycin complex. In addition, one of the non-coding SNPs was shown to alter miRNA binding sites. Characterizing nsSNPs and non-coding SNPs and their harmful effects on a protein’s structure and functions will enable researchers to understand the critical impact of mutations on the molecular mechanisms of various diseases. This will ultimately lead to the identification of potential targets for disease diagnosis and therapeutic interventions.

## 1. Introduction

The mechanistic (or mammalian) target of rapamycin (mTOR) is a serine-threonine protein kinase, located on chromosome 1p36.2. Extensive research has found that this 289-kDa protein regulates several fundamental cellular processes. These processes can be as varied as protein synthesis and autophagy [1]. mTOR is ubiquitously expressed throughout the body. In response to intracellular and extracellular signals mTOR coordinates and regulates cell growth and proliferation, cell survival, and homeostasis. On the other hand, dysregulated mTOR signaling plays a role in cancer, Alzheimer’s disease, and type 2 diabetes [2].

The kinase belongs to the PI3K-related kinase (PIKK) family and is the catalytic component of two major multi-protein complexes mTORC1 and mTORC2 which activate two downstream signaling pathways. Both complexes share mLST8 and Deptor in addition to the catalytic subunit. mTORC1 also comprises Raptor, and PRAS40 [3] and mTORC2 comprises Rictor, Sin1, and Protor proteins [4, 5]. The C-terminus of the protein contains the PI3-K kinase domain whereas its N-terminal contains at least 20 HEAT (huntingtin elongation factor-3-protein phosphatase-2A TOR1) repeats, which mediate their interaction with associated proteins [6].

mTOR has been found to be abnormally over-activated in more than 70% of cancers [7] such as breast, prostate, lung, liver, and renal carcinomas. Hyperactivated or overexpressed mTOR contributes to tumor initiation, progression, and metastasis. For instance, it has been suggested that the activation of the oncoprotein AKT relies on downstream activation of mTORC1 to drive tumorigenesis. This observation has greatly instigated the clinical development of mTOR inhibitors like rapamycin for targeted therapy of tumors [8]. The aberrant activity of the protein may result from genetic alterations as well as single nucleotide polymorphisms (SNPs).

SNPs are the most common genetic variants that are present in the human genome. They refer to a variation of a single nucleotide at a specific genomic position among individuals and occur approximately every 100–300 base pairs [9]. This type of genetic polymorphism has often been reported to be associated with a particular phenotype or disease including cancer susceptibility. For instance, one study has shown that functional SNPs of the *mTOR* gene were linked with an increased risk of esophageal squamous cell carcinoma [10]. Other studies have shown their association with gastric cancer and sporadic prostate cancer in a certain population [11, 12]. Such associations have paved the way for an increasing focus on the role of SNPs in diagnostics and risk prediction for cancer. They are now established as interesting and crucial biomarkers for cancer. In line with that, in 2016 a meta-analysis was performed covering 20 mTOR SNPs and it was found that several of the SNPs were associated with increased cancer risk like acute leukemia [13].

Missense non-synonymous SNPs (nsSNPs) have always been regarded with much concern. They lead to an amino acid change within the coding sequence and therefore can affect the protein’s activity. Determining the effect of multiple nsSNPs experimentally can be expensive and laborious. However, by using *in silico* analysis via available web-based bioinformatics tools, it is feasible and also cost-effective to study a large number of SNPs present within a particular gene. Computational genomics has made remarkable contributions to the field of disease-associated nsSNP analysis. Several algorithms have been developed for the accurate prediction of the phenotypic effects of the nsSNPs [14]. The structural behavior of the mutations is now being investigated using newer and newer approaches including molecular dynamics simulations [15–17]. Significant observations have been made from such analyses on a myriad of proteins including albinism-associated OCA2 protein [18], kinesin family protein MCAK [19], cardiomyopathy-associated MyH7 [20], etc.

The results from this study are intended to have worldwide reproducibility, require less computational time and processing memory and ensure ubiquitous dissemination. Although there are many standalone software available, they require expertise in programming languages. They are also machine-dependent. Therefore, to ensure reproducibility and repeatability, web servers were preferred to standalone computational software in this study. Here all the nsSNPs within the *mTOR* gene have been collected, screened, and analyzed to find out the most potentially harmful nsSNPs. The primary focus was given on how these genetic variations affected the protein’s structure, stability, and function as well as the physicochemical properties of the respective amino acid residues. Besides, this study also included an analysis of the non-coding SNPs that are located at the *mTOR* gene to identify any potential harmful effect of such SNPs in this locus.

Altogether 11 nsSNPs were selected as most deleterious in this study. Three of the polymorphisms (L509Q, R619C, and N2043S) were located within interacting regions of the protein, and four of the polymorphisms (K1452N, A1513D, E1610K, and R1616C) were located within a core domain indicating they might affect the protein’s native structure. All of the nsSNPs were found to decrease the protein’s stability. Three of them (R619C, A1513D, and T1977R) were found to be associated with several different carcinomas and detailed structural analyses revealed that these polymorphisms might alter C alpha distances, bond lengths, and bond angles and thus might disrupt the protein’s structure and function. In addition, one non-coding SNP (rs12139042) was found to affect miRNA binding sites which suggests that it could interfere with miRNA regulation of the mTOR protein. Overall, this analysis will provide the groundwork for further investigation into the structural and functional impact of the SNPs in the *mTOR* gene.

## 2. Materials and methods

### 2.1 Data collection

The human *mTOR* protein sequence was obtained from the UniProt database [21] (UniProt ID: P42345). The nsSNPs were retrieved from three different databases that included NCBI Short Genetic Variation database (dbSNP) (https://www.ncbi.nlm.nih.gov/snp/) [22], ClinVar (https://www.ncbi.nlm.nih.gov/clinvar/) [23] and DisGeNET (https://www.disgenet.org/) [24]. dbSNP can be considered as a catalog of any short variations in human nucleotide sequences. ClinVar only archives those human variations which have associations with different health phenotypes, with supporting data available. Similarly, the DisGeNET database accumulates information on human gene-disease associations (GDAs) and variant-disease associations (VDAs) from various disease repositories. From all three databases, only nsSNPs (missense SNPs) were filtered out. For analyzing the non-coding SNPs, the dataset was collected from the ENSEMBL database (https://asia.ensembl.org/index.html) for the respective protein ID [25].

### 2.2 Selection of the tools to determine potentially deleterious nsSNPs

To determine whether the nsSNPs will confer any deleterious effect upon mTOR protein’s 3D structure and function, in total, 10 *in silico* prediction tools were utilized. A large number of computational tools are available at present. Since no single bioinformatics tool has been demonstrated to be most effective in analyzing the harmful effects of nsSNPs, multiple tools were opted for, based on the assumption that combining scores from several tools will greatly strengthen the accuracy and reliability of the predictions. In this study two different approaches were taken into consideration while choosing the tools.

Firstly, the prediction tools were selected in a way so that they covered four different methods including sequence homology-based method, supervised learning method, protein sequence and structure-based method, and consensus-based method. The following tools were included in the study: SIFT (Sorting Intolerant from Tolerant) [26, 27], PROVEAN (Protein Variation Effect Analyzer) [28], and Mutation Assessor [29]—these three tools determined the effect of the query amino acid substitutions on the protein function on basis of sequence homology. SIFT has been widely used in computational assays carried out to determine the deleterious nsSNPs. SNAP2 and SuSPect depended on supervised learning methods. SNAP2 is based on a neural network [30], and SuSPect is based on a support vector machine [31]. PolyPhen-2 (Polymorphism Phenotyping v2) predicts the functional impact of the amino acid substitutions considering structural along with the sequence and phylogenetic information regarding the substitutions [32]. It is recommended because the quality measures are more balanced. Lastly, Meta-SNP [33] utilized a consensus score based on four different SNP impact prediction strategies including PANTHER, PhD-SNP [34], SIFT, and SNAP [33].

In another approach, two different groups of tools were included in this study. The first group of tools was used to predict the impact of the nsSNPs on the protein function and they included SIFT, PROVEAN, Mutation Assessor, CADD [35], PolyPhen2, and SNAP2. The second kind of tools was applied to assess the pathogenicity of the polymorphisms and this group included PhD-SNP, SuSPect, and PMut [36] tools.

Tools from the two different approaches have been overlapping in some cases. Since combining all these tools cover a wide range of prediction approaches, they may provide a high accuracy level in prediction. A short description along with the input parameters and accuracy rate of all the tools are presented in Table 1.

**Table 1:** List of web-based bioinformatics tools used in the study for the identification of most deleterious nsSNPs along with their description, input parameters, and accuracy rate.

Prediction tool	Description	Input parameters	Accuracy rate
SIFT	Predicts nsSNP impact on protein function based on sequence homology and the physical properties of amino acids	chromosome positions (coordinates and orientations) and alleles/ dbSNP rsIDs	76.99%
PolyPhen2	Predicts nsSNP impact on protein structure and function based on sequence, phylogenetic, and structural features	Protein identifier and amino acid substitutions	75.56%
PROVEAN	Predicts nsSNP impact on protein function based on an alignment-based scoring method	Protein query sequence and amino acid variations	79.19%
Mutation Assessor	Predicts nsSNP impact on protein function based on evolutionary conservation of the affected amino acid in protein homologs	Query protein ID and variant	78.15%
SNAP2	Predicts functional effects of nsSNPs based on a neural network involving evolutionary information, predicted secondary structure, and solvent accessibility	Query protein sequence	82%
SuSPect	Predicts phenotypic effects of nsSNPs using a support vector machine (SVM) method integrating sequence-, structure- and systems biology-based features	UniProt IDs and amino acid variations	82%
PhD-SNP	Predicts pathogenicity of nsSNPs by an SVM-based method using sequence and profile information	Query protein sequence and amino acid variations	78%
PMut	Predicts pathology of mutations based on a neural network and sequence conservation information	Protein UniProt ID and amino acid variations	80%
CADD	Predicts deleteriousness of nsSNPs using a machine learning model based on sequence context, gene model annotations, evolutionary constraint, epigenetic measurements, and functional predictions.	Chromosomal coordinates and allele information	-
Meta-SNP	Detects disease-associated nsSNPs by integrating four methods: PANTHER, PhD-SNP, SIFT and SNAP	Query protein sequence and amino acid variations	79%

### 2.3 Evolutionary conservation, surface accessibility, and post-translational modification (PTM) analysis

The degree to which an amino acid position is evolutionarily conserved, reflects its structural and functional importance. Therefore, the conservancy of each position in the amino acid sequence of the mTOR protein was analyzed by the ConSurf server (https://consurf.tau.ac.il/) [37]. This tool estimates the evolutionary rate of each amino acid position based on the phylogenetic relations between homologous sequences. The software can correctly distinguish genuine sequence conservation from other conservation that can result from a short evolutionary time. In this study, the mTOR protein sequence was analyzed against the UNIREF-90 protein database using the HMMER homolog search algorithm with a 0.0001 E-value cut-off and 3 HMMER iterations.

To determine the surface accessibility of each amino acid residue of mTOR protein, NetSurfP-2.0 (https://services.healthtech.dtu.dk/service.php?NetSurfP-2.0) was used [38]. This web tool is sequence-based and uses neural networks to predict several local structural features including solvent accessibility, secondary structure, and structural disorder for each residue of the query sequences.

Post-translational modifications within a protein molecule are also crucial. They introduce new functionalities and exert control over protein structure and function by modulating intra- and intermolecular interactions. In this study, the web-based tool MusiteDeep (https://www.musite.net/) was applied to predict potential PTM sites and corresponding probable modifications in the mTOR protein sequence [39]. Rather than predicting only a single type of PTM, this web server provides a deep-learning framework for general protein PTM site prediction and visualization using raw protein sequences as input.

### 2.4 Determining impact on protein stability

Whether the amino acid substitutions affected mTOR protein’s stability, two different web tools were applied: I-mutant 3.0 (http://gpcr2.biocomp.unibo.it/cgi/predictors/I-Mutant3.0/I-Mutant3.0.cgi) and MUpro (http://mupro.proteomics.ics.uci.edu/). I-mutant 3.0 predicts the effects based on a support vector machine [40]. MUpro incorporates a set of machine learning programs to determine the effect of SNPs [41]. In I-Mutant, the query protein sequence and the amino acid variation were given as input. Using the sequence information, the change in Gibbs free energy change (ΔΔG) and its sign were predicted for the single point mutations. In MUpro, the same predictions regarding protein stability including the value of energy change (delta delta G) and the sign of energy change were made. In neither of the tools, the tertiary structure of the protein was provided however for MUpro, it has been shown that the prediction accuracy using sequence information alone (84.2%) is comparable to that of using tertiary structures (84.5%) [42].

### 2.5 Predicting phenotypic effects of the nsSNPs

To analyze the structural and functional effects of the point mutations, the HOPE (Have (y)Our Protein Explained) (https://www3.cmbi.umcn.nl/hope/) [43] server was utilized. Being a next-generation web application, this automated mutant analyzer creates a report on each mutation illustrating the effects of the mutation on the protein’s size, charge, bonding pattern, and interaction with other molecules. For this, the protein sequence, the positions of the amino acid alterations, and the altered residues were submitted as input. Another web tool MutPred2 (http://mutpred.mutdb.org/) was also utilized for this purpose. MutPred2 predicts the phenotypic impact of the amino acid alterations based on a neural-network method [44]. The impacts may include a protein’s stability and structure disruption, macromolecular binding disruption, PTM site excision, etc. which can lead to major altered phenotypic properties of a protein. In this server, the fasta sequence of mTOR protein and the amino acid variations of interest were given as input, and the P-value threshold was set at default (0.05).

### 2.6 Predicting mutation clusters

Mutation3D (http://www.mutation3d.org/about.shtml) was applied to find out whether the amino acid substitutions belonged to any cluster that could reveal any functional hotspots of mutations [45]. Based on a complete-linkage clustering algorithm, this tool can effectively identify clusters of amino acids on protein models and structures.

### 2.7 Predicting protein-protein interaction

The knowledge of the interaction partners for a protein is crucial to understanding its function, structure, molecular action, and regulation. the STRING (https://string-db.org/) database was utilized to find out with which proteins mTOR interacts [46]. The protein was searched by its name and the search was run with the highest confidence score.

### 2.8 Predicting association of the nsSNPs with cancer susceptibility

Several different tools were applied to determine the oncogenic potential of the selected somatic point mutations including CScape (http://cscape.biocompute.org.uk/), CBioportal (https://www.cbioportal.org/), and canSAR Black (https://cansarblack.icr.ac.uk/). CScape depends on a statistical approach to identify cancer-driving mutations with a 91% balanced accuracy in coding regions of the genome [47]. In the web server, the list of mutations was entered using the format chromosome, position, reference, and mutant, and predictions were given as p-values in the range between 0 and 1. Values above 0.5 are predicted to be deleterious, while those below 0.5 are predicted to be neutral or benign. The next server was cBioPortal which is a repository with extensive data on the molecular landscape of different types of cancer [48]. In this platform, the *mTOR* gene was queried against the curated set of non-redundant studies including 190 studies and 59519 samples. The resulting missense mutations that were found in patient samples with different types of cancer were retrieved from the analysis. Another resource that was used for retrieving known associations between the SNPs with cancer studies was canSAR Black. It is a multidisciplinary knowledge base for translational research on cancer [49]. In this server, mTOR protein was searched by its name and from the results, its association with different cancer types was collected. Each cancer data showed mTOR mutations found for that particular cancer type and from there the filtered SNPs were checked whether they were present in the selected cancer sample.

### 2.9 Protein 3D modelling and structural analysis

The 3D structures of the wild-type protein and proteins with the mutated amino acids were modelled in the homology modelling server- SWISS-MODEL (https://swissmodel.expasy.org/) [50]. In this server’s workspace, the amino acid sequence of mTOR protein was given as input and the protein’s structure was computed in an automated mode through four steps. The steps included identification of structural templates, followed by alignment of the target sequence and template structure, and finally model-building and model quality evaluation. The automatic mode identifies suitable templates based on BLAST [51], and HHblits [52]. The quality of the predicted 3D structures was assessed by the SWISS-MODEL structure assessment tool and SAVES v6.0 server (https://saves.mbi.ucla.edu/) [53].

After all the mutated models as well as the wild-type protein model were predicted and verified, they were analyzed in TM-align [54] and PyMOL for their template modeling score (TM-score) and root mean square deviation (RMSD) value. TM-score provides a means to assess the topological similarity of protein structures allowing one to determine whether two protein structures share the same fold or not. And PyMOL is an open-source tool through which the 3D structure of molecules can be visualized. Afterwards, UCSF Chimera was used [55] to visualize the H bonds. UCSF Chimera allows for interactive visualization and analysis of molecular structures and related data. Finally, the homology predicted structures were also analyzed by the Missense 3D tool which can determine any structural damage caused by an amino acid substitution [56].

### 2.10 Analysis of 5´ and 3´ UTR non-coding SNPs

The non-coding SNPs located at the 5´ and 3´ UTRs were filtered out from the Ensemble database with a global minor allelic frequency (MAF) value between 0.05 and 0.5 [25]. Later the SNPs were run in RegulomeDB [57] to assess their functional consequences. RegulomeDB utilizes multiple high-throughput, experimental data sets from GEO, ENCODE, and other sources to identify non-coding SNPs with potential regulatory roles. Finally, PolymiRTS (Polymorphism in microRNAs and their Target Sites) database was searched to find out whether any of the non-coding SNPs was located in microRNA (miRNA) seed regions and miRNA target sites [58].

**Fig 1:**
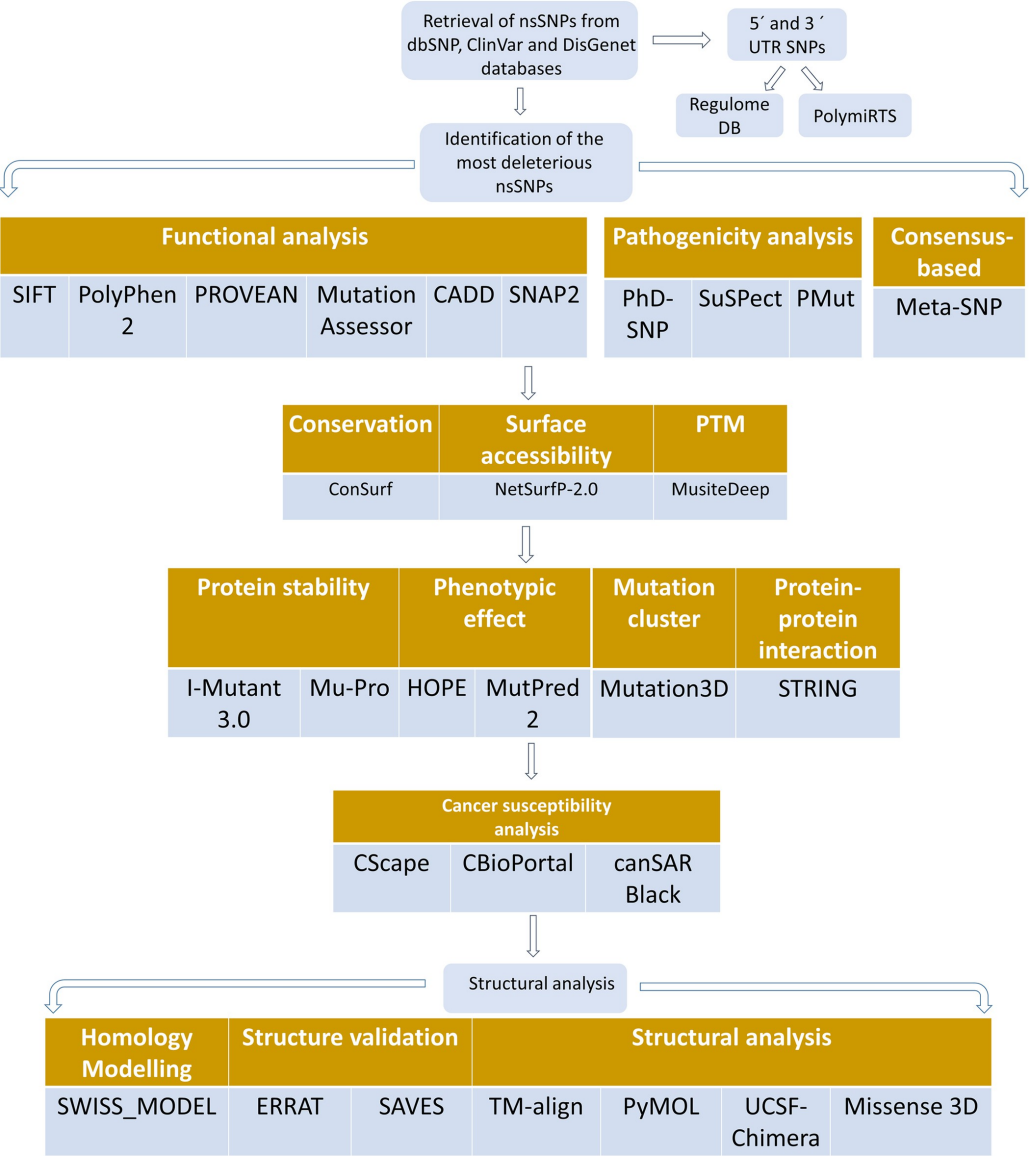
A flowchart depicting the entire study plan along with the tools that have been applied in the study.

The entire study design including the various tools used is shown in Fig 1.

## 3. Results

### 3.1 Retrieval of SNPs from different databases

**Fig 2:**
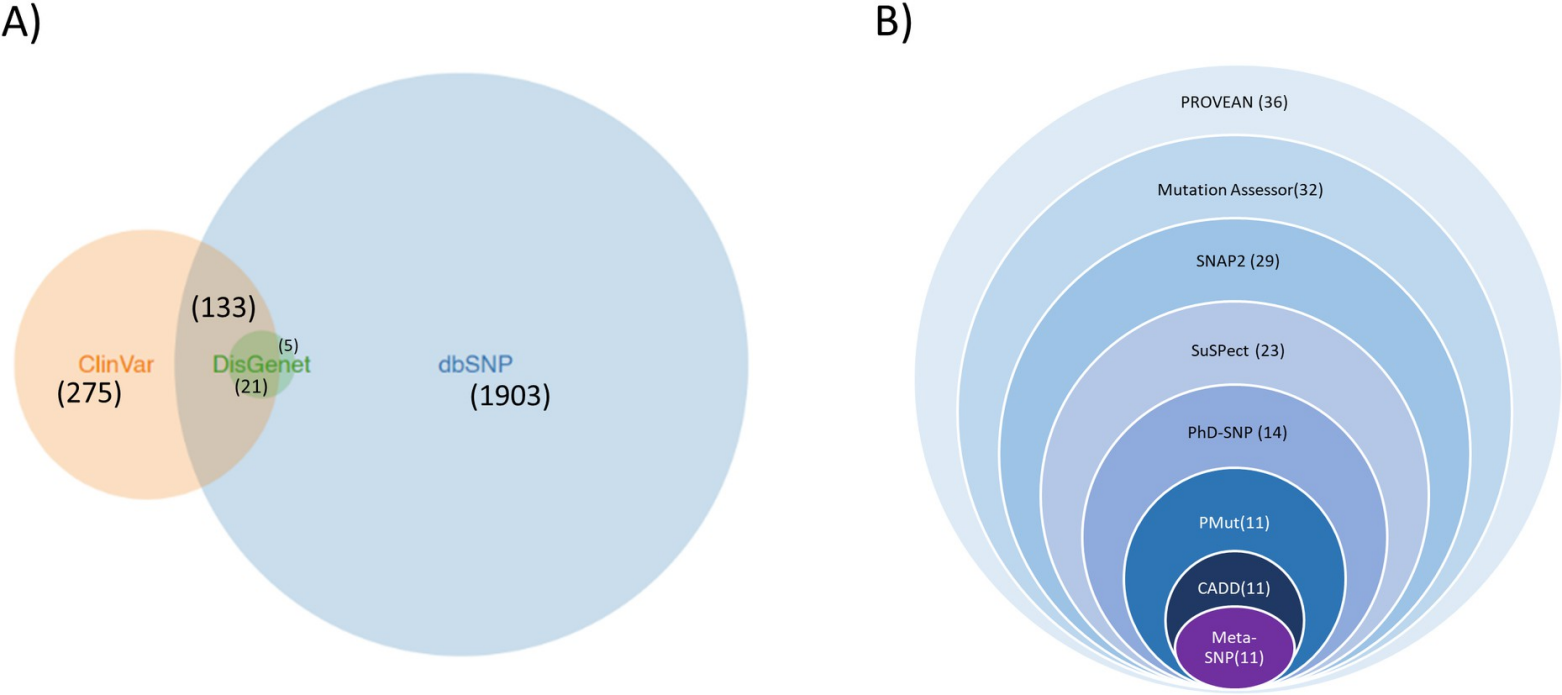
Retrieval and Identification of most deleterious nsSNPs. A) Retrieval of nsSNPs from three different databases: dbSNP, ClinVar, and DisGenet. The numbers in brackets indicate how many nsSNPs were retrieved from each database and how many overlapped between different databases. B) Identification of the most potentially harmful and pathogenic nsSNPs through bioinformatics analyses. The numbers in the bracket after each tool represent how many deleterious nsSNPs resulted from the previous analysis overlapped with the results from the current analysis. After 10 different tools were applied, eleven nsSNPs were selected as potentially most harmful.

The NCBI dbSNP database [22] contained 60975 SNPs for the *mTOR* gene including 57529 variations in the intronic region and 3446 variations in the coding sequence. Out of the SNPs in the coding sequence, 1903 SNPs were classified as non-synonymous SNPs and were retrieved from the database. Another 408 variations were found documented in ClinVar [23] and 26 in DisGenet [24]. The ones found in DisGenet were all found to be redundant, when compared to dbSNP retrievals. Out of these 26 SNPs, 21 also overlapped with ClinVar IDs. However, 275 SNPs from ClinVar were found to be novel SNPs (Fig 2A). Therefore, in total 2178 nsSNPs and their information were gathered for the *in-silico* analysis.

### 3.2 Selection of the most deleterious nsSNPs

Using SIFT algorithm [26, 27] the effects of the nsSNPs were determined and 227 SNPs with rsIDs resulted from the analysis. Out of these227 SNPs, 34 SNPs were found to confer deleterious effects on protein function as they scored below or equal to 0.05 in a range between 0 and 1. The other 1676 nsSNPs could not be found from the results of the SIFT analysis. On the other hand, from the 275 nsSNPs from the ClinVar database, 36 SNPs were predicted non-synonymous. Out of those 36, nine SNPs were predicted to be damaging by SIFT. Therefore, from SIFT analysis, in total 63 nsSNPs were found to be deleterious.

On the other hand, PolyPhen-2 [32] predicted 938 of the nsSNPs to have deleterious effects, and 38 of these SNPs overlapped with SIFT findings. The probability score that is given by PolyPhen-2 ranges between 0.0 (tolerated) and 1.0 (deleterious). Values close to 0 indicate that the variant is benign and values closer to 1.0 indicate that the substitution is most probably damaging.

Of these 38 nsSNPS, 36 were found to be deleterious by PROVEAN [28] score as well, since their scores were below or equal to the cut-off value of -2.5. The score values ranged between -7.829 and -2.528. Afterwards, these 36 nsSNPs were analyzed by the rest of the tools (shown in S1 Table), and only those SNPs that were predicted to have harmful effects by every tool were analyzed further.

When these nsSNPs were analyzed by Mutation Assessor [29], it was found that the resulting amino acid substitutions exhibited three different functional impacts- low, medium, and high. Only those which made a medium and high impact on the protein’s function were selected from this tool and 32 nsSNPs fell into the selected category. The effect of the amino acid variations was also checked in SNAP2 [30] and the resulting scores ranged between -100 strong neutral prediction to +100 strong effect prediction. It showed that 29 of these mutations were likely to alter the native protein function. These 29 mutations have been labeled as ‘effect’ while the other seven have been classified as neutral. When these amino acid substitutions were analyzed by SuSPect [31], a different range of scores than SNAP2 was obtained. Scores were predicted between 0 and 100 and were also color-coded according to deleteriousness. One end of the spectrum was blue representing neutral and the other end was red representing disease-causing. It was found that 30 of these polymorphisms were predicted to be disease-causing variants as their score crossed the cut-off value of 50.

The deleteriousness of the single nucleotide variants was also cross-checked by PhD-SNP [34] and PMut [36] webservers. Results from PhD-SNP showed that 21 of these mutations were disease-related while the other 15 were neutral. Analyses from PMut showed that 25 of these mutations were disease related as their scores crossed the cut-off value of 0.5. The remaining 11 mutations were neutral.

When the chromosomal coordinates of these nsSNPs were analyzed by CADD (Combined Annotation-Dependent Depletion) [35], the scaled C-score (PHRAD) of all the polymorphisms was greater than 20. It indicated these were among the 1% most deleterious substitutions for the human genome. Lastly, Meta-SNP [33] was applied to again predicting any associations of the SNPs with disease. This tool integrates four existing methods: PANTHER, PhD-SNP, SIFT, and SNAP and this was particularly useful as the evolutionary analysis of the nsSNPs could not be done individually by PANTHER. Since no PANTHER family for the input sequence could be found, the variations were not scored. Meta-SNP analysis revealed that 28 of these nsSNPs were disease-related Eleven mutations were found to be potentially harmful by every tool (Table 2) while the rest were predicted to be neutral by at least one of the tools shown in S1 Table. Hence for further analysis, these eleven polymorphisms were selected which were L509Q, R619C, D944V, Y1151C, R1161G, K1452N, A1513D, E1610K, R1616C, T1977R, and N2043S. Fig 2B shows how stepwise filtration through eight different bioinformatics tools after SIFT and PolyPhen2 led to the selection of these eleven most deleterious nsSNPS.

**Table 2:** The predictions and scores of the most deleterious nsSNPs determined by 10 bioinformatics tools along with their dbSNP IDs if available.

AA variation	dbSNP ID	SIFT Score	PolyPhen2	PROVEAN	Mutation Assessor	SNAP2	SuSPect	PhD-SNP	PMut	CADD (PHRAD)	Meta-SNP (score)
L509Q	28730691	0.023	0.997	-4.7	medium	20	84	disease	0.58	29.6	0.53
R619C	199712134	0	1	-7.8	high	45	97	disease	0.76	31	0.83
D944V	-	0	0.977	-5.9	medium	54	58	disease	0.59	28.9	0.75
Y1151C	151082401	0.017	0.983	-6.3	Medium	57	87	disease	0.64	24.8	0.78
R1161G	202197441	0.002	0.976	-6.3	medium	46	72	disease	0.64	25.8	0.69
K1452N	-	0	0.981	-4.1	medium	21	57	disease	0.80	23.5	0.68
A1513D	374529391	0.001	0.999	-4.1	medium	40	86	disease	0.75	28.6	0.70
E1610K	199612643	0.022	0.996	-3.5	medium	37	43	disease	0.59	32	0.55
R1616C	17848545	0	1	-5.4	medium	20	83	disease	0.67	32	0.69
T1977R	-	0	1	-5.2	medium	59	66	disease	0.80	28.9	0.72
N2043S	371511548	0.005	1	-3.9	medium	16	75	disease	0.75	25.7	0.56

### 3.3 Evolutionary conservancy and surface accessibility show highly conserved and functional SNP sites

**Fig 3:**
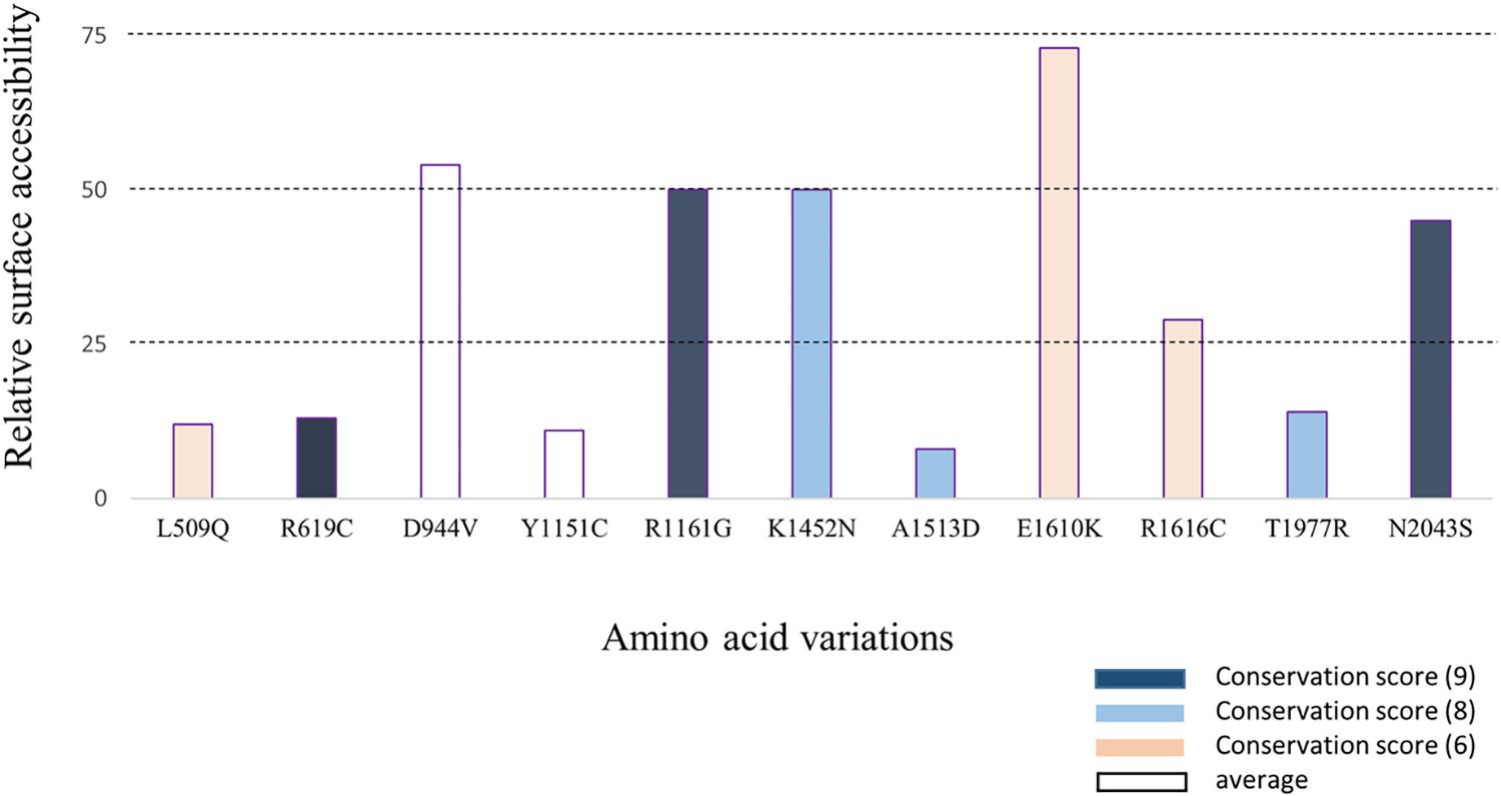
Conservancy and solvent accessibility analysis of selected amino acid positions within mTOR protein. Position of the most deleterious SNPs along with their relative surface accessibility predicted by NetSurfP-2.0 with a threshold of 25% which represents SNPs having *>*25% RSA are predicted to be exposed on the protein surface. The conservation state of the SNP positions predicted by ConSurf is represented with color codes.

ConSurf estimates the degree of conservation of the amino-acid residues among their close sequence homologs and thus identifies the functional regions of a protein. For this analysis a neighbor-joining phylogenetic tree was constructed using multiple sequence alignment (MSA) data that was built by the default program, MAFFT (Multiple Alignment using Fast Fourier Transform). Also, a position-specific conservation score on a scale of 1–9, where 9 indicates the most conserved region and 1 indicates the most variable region was provided for each residue (Fig 3). It was found that three of these positions (R619C, R1161G, and N2043S) were highly conserved and scored 9 and another three positions (K1452N, A1513D, and T1977R) scored 8. The rest of the positions showed an average level of the conservancy. Due to their high conservancy, R619C, R1161G, K1452N, and N2043S- these four sites were also marked as functional sites of mTOR protein.

NetSurfP-2.0 analysis revealed the relative surface accessibility of each amino acid residue of mTOR protein by assigning a percentage score to them. If the score was more than 25%, it implied that the amino acid residue was exposed. In this work, D944V, R1161G, K1452N, E1610K, R1616C, and N2043S positions were all predicted to be in an exposed position (Fig 3). Interestingly, although R619C was predicted to be buried by NetSurfP-2.0 analysis, ConSurf predicted the residue to be exposed. The rest of the positions were predicted to be buried by both analyses. Through using MusiteDeep web tool, probable PTM sites were also predicted in the mTOR protein. However, none of the most deleterious nsSNP positions correlated with a potential PTM site.

### 3.4 SNPs bring potential changes in protein stability and structure

When the nsSNPs were analyzed by I-Mutant 3.0 and MUpro servers, it was found that all of them decreased mTOR protein’s stability as represented by their score which was less than 0 for every mutation. The free energy change (ΔΔG) values along with their signs predicted by the two servers are presented in Table 3. According to I-Mutant 3.0, when the ΔΔG value comes below -0.5 Kcal/mol, it indicates the said mutation can largely destabilize the protein. In this study, such destabilizing values were computed for L509Q, R619C, Y1151C, R1161G, A1513D, R1616C, and N2043S positions.

**Table 3:** The effect of the nsSNPs on mTOR protein’s stability as determined by I-Mutant 3.0 and MUpro and on structural features of the protein as determined by HOPE.

	I-Mutant 3.0	MUpro	Summary of HOPE report				
Amino acid variation	ΔΔG value (Kcal/mol)	ΔΔG value (Kcal/mol)	Affects size and charge	Affects hydrophobicity	Disrupt hydrogen bond/Salt bridge	Interferes with protein function	Interferes with other protein interaction
L509Q	-1.86	-1.84	Affects size	Yes	No	Yes	No
R619C	-0.93	-0.66	Yes	Yes	Disrupts hydrogen bond	Yes	Yes
D944V	-0.26	-0.55	Yes	Yes	Disrupts salt bridge	Yes	Yes
Y1151C	-1.15	-0.12	Affects size	Yes	Disrupts hydrogen bond	Yes	No
R1161G	-1.46	-1.61	Yes	Yes	Disrupts salt bridge	Yes	Yes
K1452N	-0.17	-0.91	Yes	No	Disrupts hydrogen bond and salt bridge	Yes	Yes
A1513D	-0.85	-0.99	Yes	Yes	No	Yes	No
E1610K	-0.29	-1.25	Yes	No	Disrupts salt bridge	Yes	Yes
R1616C	-0.72	-0.56	Yes	Yes	Disrupts hydrogen bond and salt bridge	Yes	Yes
T1977R	-0.23	-0.67	Yes	Yes	Disrupts hydrogen bond	Yes	No
N2043S	-0.53	-1.12	Affects size	Yes	Disrupts hydrogen bond	Yes	Yes

Afterwards, the nsSNPs were run through project HOPE which aims at providing insight into the structural effects of a mutation including amino acid size, charge, hydrophobicity, spatial structure, and function.

**Fig 4:**
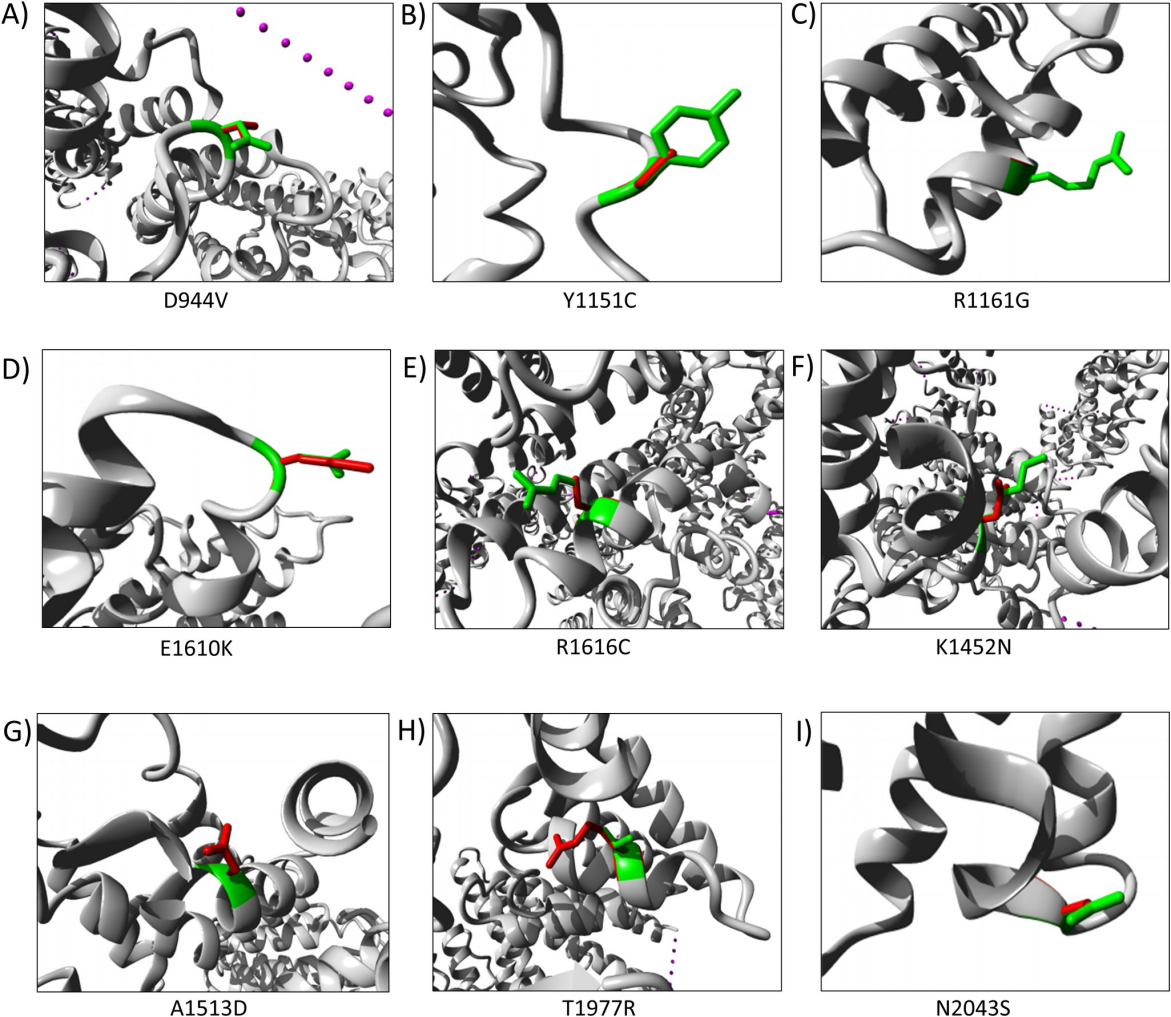
Structural images of the nine nsSNPs created by the HOPE report. Images of the modified structures of mTOR protein were generated using Project HOPE. The whole protein is pictured in grey colour, the side chain of the wild-type residue is indicated in green and of the mutant residue is shown in red. In A, B, C, E, F, and I, the mutated residue which is in red is smaller than the wild-type residue presented in green. On the other hand, in D, G, and H the mutant residues are bigger than the wild type residues. All of these structural changes will disrupt the protein’s shape, conformation, and function. Note: (A) D944V (B) Y1151C (C) R1161G (D) E1610K (E) R1616C (F) K1452N (G) A1513D (H) T1977R and (I) N2043S.

The detailed analysis for each of the substitutions is presented in Table 3. From the reports, it could be seen that every amino acid substitution affected the protein’s size and, in some cases, charge also. Among these 11 sites, there were 8 sites with changes in the charge: 4 changed from positive to neutral, 1 changed from negative to neutral, 1 changed from neutral to positive, 1 changed from neutral to negative and 1 changed from negative to positive. In the case of R1161G, K1452N, and R1616C, the wild-type residues lost their charges. It was predicted that such variations could affect the protein’s interaction with other molecules. On the other hand, A1513D and T1977R substitutions introduced a negative and a positive charge respectively, in a buried residue within the protein which can lead to protein folding problems. To be folded in the minimum-energy configuration, the hydrophobic residues of a protein usually remain together in the core while the hydrophilic ones are exposed to the surface. For nine of the substitutions, HOPE could generate structural images showing both wild type and the altered amino acid residue at the particular protein site and they are shown in Fig 4.

In addition, some of the amino acid positions including Y1151C, A1513D, and T1977R are within the core domain of the protein. Changes at these residues were predicted to affect the core structure of the protein. Particularly with Y1151C, it was predicted that such a substitution would create an empty space within the core. In this case, the mutant residue became smaller than the wild-type residue and that might be too small to make multimer contacts. Moreover, conservancy analysis showed that at 619, 944, and 1610 positions, although the wild-type residues were not conserved, the mutant ones were also not commonly observed in the homologous sequences. Therefore, this analysis also predicted these mutations to be probably damaging to the protein.

Certain effects of two amino acid positions were mentioned in the HOPE report. R1161G mutation introduced a glycine at this position and glycines are very flexible. This mutation was therefore predicted to disturb the required rigidity of the protein at this position. Another was N2043S substitution, which is located in a region that interacts with the FKBP1A/rapamycin complex. This amino acid change could disturb this region and interfere with this interaction.

When the mutations were next analyzed by the MutPred2 server, 9 out of 11 nsSNPs scored higher than 0.8 which indicated that they had high pathogenic properties. In this server, it was predicted whether these mutations caused any gain of intrinsic disorder. Additionally, whether they caused structural modifications and DNA binding alterations were also predicted with P-value and Probability value. It was found that two of the mutations A1513D and T1977R caused loss of ubiquitination, while R619C caused loss of acetylation. In addition, several of the alterations including K1452N, A1513D, R1616C, and T1977R led to altered transmembrane properties. Similarly, E1610K and R1616C variations caused an altered disordered interface. The details of the MutPred2 results are given in the S2 Table.

Lastly in this section, mTOR protein was also analyzed by mutation 3D web server. All 36 nsSNPs were given as input and it was found that amino acid residues at positions 1609, 1610, and 1616 belonged to a cluster with a P-value of 6.25e-02.

### 3.5 Determining major interacting molecules of the mTOR protein

From the STRING network, the 10 major interacting molecules with mTOR protein were revealed which included Eukaryotic translation initiation factor 4E-binding protein 1, GTP-binding protein Rheb, Regulatory-associated protein of mTOR (RPTOR), DEP domain-containing mTOR-interacting protein (DEPTOR), Rapamycin-insensitive companion of mTOR (RICTOR), Target of rapamycin complex 2 subunit MAPKAP1, Serine/threonine-protein kinase ULK1, Ras-related gtp-binding protein c/d (RRAGC), Ribosomal protein S6 kinase beta-1 and Tuberin, a tumor suppressor protein (S1 Fig). Since several of the nsSNPs were predicted to interfere with the interaction of mTOR with other molecules, the function of these interacting molecules could also get affected by these amino acid alterations.

### 3.6 The association between the high-risk nsSNPs with cancer susceptibility

The oncogenic potential of the point mutations was first assessed by CScape. The results showed that L509Q, D944V, A1513D, E1610K, and T1977R were deleterious, predicted with the highest confidence score. Next, these mutations were also analysed by the CBioPortal server over 190 non-redundant curated sets of studies. The position of each of the missense or non-synonymous SNPs was checked and it was found that several of these mutations were present in different cancer patients’ samples. The detailed results are shown in Table 4.

**Table 4:** Association study of the high-risk nsSNPs with different types of cancer obtained from Cscape, cBioPortal and canSAR Black databases.

Amino acid variation	Cscape	cBioPortal	canSAR Black
L509Q	0.92		
R619C	0.86	Renal clear cell carcinoma, melanoma, uterine endometrioid carcinoma	Uterine endometrial
D944V	0.91		
Y1151C	0.88	Lung adenocarcinoma	Lung
R1161G	0.85		Brain
K1452N	0.58		Kidney
A1513D	0.91	Uterine endometrioid carcinoma	Uterine endometrial
E1610K	0.92	Prostate adenocarcinoma, colorectal adenocarcinoma	
R1616C	0.78	Cutaneous melanoma	
T1977R	0.95	Acute lymphoid leukemia, lung adenocarcinoma, renal clear cell carcinoma, colorectal carcinoma	Lymphoma, Leukemia, Uterine endometrial, Kidney, Prostate
N2043S	0.86		

(Yellow values denote low-confidence oncogenic predictions and red values denote high-confidence oncogenic predictions made by Cscape)

It could be seen that in some cases the results from CBioPortal and CanSAR Black overlapped with each other while in other cases novel associations were revealed. Among the cancer types, uterine endometrial, melanoma, renal clear cell carcinoma, kidney, prostate, and lung cancer, each was found to be linked with two different mutations. Interestingly, L509Q and D944V substitutions were not found to be associated with any cancer study although Cscape predicted these mutations to be oncogenic with high confidence.

### 3.7 Structural analyses of the protein mutations

The 3D structure of the mTOR protein was predicted in the SWISS-Model workspace using 6zwm as a template [59]. Also, the protein’s structure containing each of the 11 amino acid alterations was predicted individually. After the predictions were done, the quality of the protein models was checked using the Ramachandran plot, QMEAN score, and MolProbity score computed from the SWISS-MODEL structure assessment tool and ERRAT score from the SAVES server. The results are shown in Table 5. In an ideal case, Ramachandran favored region of a protein structure should be greater than 98%. All the predicted models in this study including the wild-type and the mutated ones had 92–93% residues in the Ramachandran favored region. QMEAN Z-scores reveal the degree of nativeness of a given protein structure with scores around 0.0 reflecting a native-like structure and scores below -4.0 indicating a model with low quality. In this study, all the models had a Z score of around -1 or -2. Another assessment score was MolProbity Score which is also a protein quality score that refers to the crystallographic resolution at which such a quality would be expected. This score should be as low as possible. The last tool that was used to cross-check the quality of the protein structures was the ERRAT score. ERRAT is considered an overall quality factor for non-bonded atomic interactions, with higher scores indicating higher quality. If a protein model scores more than 50, it is regarded as a high-quality model [60], and it could be seen that in this study all the predicted models scored more than 89. The Ramachandran plots generated by PROCHECK for all the mutant structures as well as the wild-type model are shown in S2–S13 Figs.

**Table 5:** Structure validation of the 3D protein models of mTOR protein and TM-score and RMSD values of mutated mTOR and wild-type mTOR.

Protein model	Template and sequence identity (%)	SWISS Model Ramachandran favored region	QMEAN score	MolProbity score	ERRAT score	TM-score	RMSD value
Wt	6zwm.1.A (100)	92.93%	-2.01	1.33	92.0821	-	-
L509Q	6zwm.1.A (99.96)	93.56%	-1.96	1.27	92.4473	0.9747	0.045
R619C	6zwm.1.A (99.96)	93.13%	-2.02	1.45	91.63	0.9768	0.094
D944V	6zwm.1.A (99.96)	93.13%	-2.05	1.3	92.719	0.9728	0.011
Y1151C	6zwm.1.A (99.96)	93.13%	-1.99	1.31	91.7826	1.0000	0.087
R1161G	6zwm.1.A (99.96)	92.93%	-2.04	1.3	92.1816	1.0000	0.052
K1452N	6zwm.1.A (99.96)	92.89%	-2.06	1.39	91.9983	1.0000	0.047
A1513D	6zwm.1.A (99.96)	93.20%	-2.05	1.41	89.8113	0.98	0.139
E1610K	6zwm.1.A (99.96)	92.97%	-2.03	1.33	92.1207	1.0000	0.011
R1616C	6zwm.1.A (99.96)	92.97%	-2.10	1.43	92.4204	1.0000	0.062
T1977R	6zwm.1.A (99.96)	93.44%	-2.10	1.37	90.3725	0.9800	0.123
N2043S	6zwm.1.A (99.96)	93.40%	-2.22	1.39	91.288	0.9671	0.078

Structural comparisons were needed to be performed to find out whether the mutated protein structures differed from the wild-type protein structure. For this purpose, two different scores were analyzed, and the results are also shown in Table 5. TM-align performs a sequence-independent structural comparison and the result, given by the TM-score scales the topological similarity between two structures. This score ranges between 0 and 1, where 1 indicates a perfect match between the wild-type and the altered structures. The second score was the RMSD (Root-mean-square deviation) value which reflects the average deviation between the corresponding atoms of two proteins. Smaller values indicate higher similarity between two structures. It can be seen from Table 5 that in the case of R619C, A1513D, and T1977R substitutions, when the mutated structures were compared with the wild-type protein model, the TM-score came to be less than 1 although the RMSD values were similar. The TM score shows that the particular mutations might lead to significant changes in the mTOR protein’s structure. The superimposed structures of these three mutants over wild-type mTOR are shown in S14–S16 Figs.

Based on this observation, the predicted 3D structures of the mTOR protein containing the following mutations: R619C, A1513D, and T1977R were visualized in PyMOL and Discovery studio. Particular focus was given to how these mutated residues differed from the wild-type version in their interaction with their neighboring residues in terms of bond angle, C alpha distance, and the number of hydrogen (H) bonds. When the angle between 619R with adjacent residues at 618 and 620 were measured, it was found to be 93.5 Å with respect to O atoms, and when the wild-type residue was replaced by Cysteine, the angle changed to 97.2 Å. When alanine was mutated to aspartic acid at position 1513, the angle changed from 90.6 Å to 90.5 Å. And when threonine changed to arginine at the 1977 position, the angle decreased from 81 Å to 78.3 Å. In all three cases, the structures of the mutated proteins were markedly changed at the site of the point mutation. Similarly, when the distance between the C alphas of the tripeptides containing the altered amino acid in the middle, was measured, all three showed changed distances. In the case of R619C mutation, the C alpha distance between 618 and 619 positions increased from 3.0 Å to 3.1 Å while the distance between 619 and 620 residues decreased from 3.2 Å to 3.1 Å. In the case of A1513D substitution, the distance increased between 1512 and 1513 positions from 3.1 Å to 3.2 Å. Again, for T1977R, the distance increased between 1977 and 1978 residues from 3.1 Å to 3.2 Å. When the C alpha distances changed due to these alterations, the dihedral angles also got affected which would ultimately affect the shape of the alpha-helices.

Using UCSF Chimera, the hydrogen bond interactions of these three residues were also visualized. In every case, the number of H bond interactions remains unchanged although the distance changed. The detailed result is shown in Table 6.

**Table 6:** Alteration of H bond interactions and their lengths due to amino acid substitutions.

Protein model	Wild type		Altered	
	H bond	Bond length (Å)	H bond	Bond length (Å)
R619C	R619-H615	3.294	C619-His615	3.238
	R619-A623	3.06	C619-A623	2.964
A1513D	A1513-Q1509	3.006	D1513-Q1509	3.073
	A1513-A1517	2.897	D1513-A1517	2.810
T1977R	T1977-I1973	3.005	R1977-I1973	3.113

The predicted structures of all the mutant proteins were also analyzed in Missense 3D and interestingly for the same three amino acid variations, structural damage was predicted. For R619C polymorphism, it was predicted that since a buried charged residue was getting replaced with an uncharged residue, it may disrupt a wild-type salt bridge. The salt bridge was between the NE atom of ARG 619 and the OD1 atom of ASP 666. For A1513D substitution, a protein folding problem was predicted as a buried hydrophobic residue was getting replaced with a hydrophilic residue and thus a charge was introduced into a buried residue. Finally, for T1977R substitution, a change was predicted between the buried and exposed state of the target variant residue where the wild-type threonine was buried and mutant arginine was exposed.

### 3.8 Analysis of the non-coding SNPs at the 5´ and 3´ UTRs

Only three non-coding SNPs could be retrieved from the Ensemble database with a global minor allelic frequency (MAF) value ranging between 0.05 and 0.5. In RegulomeDB database analysis, one (rs2295079) out of the three SNPs scored 0.88219 and ranked 2b which indicated that several datatypes including TF binding, any motif, DNase Footprint, and DNase peak were available for the chromosomal position. The probability score that was close to 1 also suggested that the respective SNP would most likely be a regulatory variant. Afterwards, the three SNPs were checked in PolymiRTS Database 3.0 and it was found that rs12139042 has two alleles C and T in the database with the functional classes of D and C, respectively in all their target sites with negative context scores. Class D denoted that the derived allele disrupted a conserved miRNA site while class C denoted that the derived allele created a new miRNA site.

## 4. Discussion

As a major regulator of cell growth and proliferation, the mTOR signaling pathway influences several different key processes in the body including apoptosis, growth, and autophagy of cells [61]. The protein has also been found to be aberrantly expressed in multiple diseases like tumorigenesis, arthritis, and osteoporosis [62]. One of the causal factors for such abnormal expression of a protein lies within its SNPs. SNPs are widespread in the human genome and can vary from one person to another. While they may change the encoded amino acids, they can also be located within the non-coding regions of a gene. Unraveling the relationship between genetic variations like SNPs and their functional and structural impact on a protein may lead to potential biomarkers for disease diagnosis and prognosis. Accordingly, several studies have identified nsSNPs within the *mTOR* gene to be associated with acute leukemia, colon cancer, gastric cancer, and many other types of cancer [13] as well as with the risk of diabetes mellitus [63]. However, the entire landscape of SNPs located in the *mTOR* locus and their potential effects on the protein’s structure and function have not yet been characterized. In the present study, a comprehensive *in silico* analysis has been performed using computational tools to identify potentially deleterious coding and non-coding SNPs within the target gene.

Several different tools were combined for the initial screening of the most deleterious nsSNPs to ensure a robust and accurate prediction with increased reliability. Some tools including SIFT, Mutation Assessor, and PROVEAN based their prediction on parameters like sequence homology, evolutionary conservation, and physical properties of the amino acids. Others like SNAP2 and PolyPhen2 relied on machine learning methods to predict the structural and functional impact of the variations. Some additional tools including PhD-SNP, SuSPect, and PMut were also included in the analysis to directly assess whether the polymorphisms were associated with pathogenicity. In total, 11 nsSNPs were selected as most deleterious as they were predicted to be high-risk by all the SNP prediction algorithms applied in this study.

**Fig 5:**
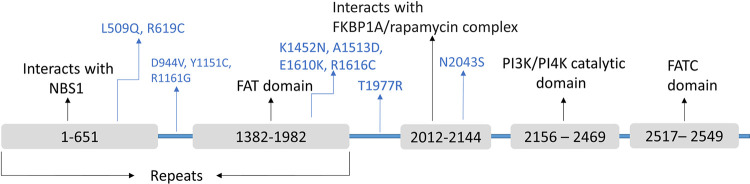
Domains and interaction sites within the mTOR protein. The positions of the final 11 nsSNPs are shown within mTOR protein with respect to the NBS1 interacting domain, FAT domain, PI3K/PI4K catalytic domain, and FATC domain. The domains are shown in grey horizontal bars and the nsSNPs are depicted by blue arrows. The exons (58 exons) are shown by green vertical bars while the introns are shown by green horizontal lines. The scaling of the exon positions and the domains is approximate.

The conservancy analysis revealed six of the selected SNPs (R619C, R1161G, K1452N, A1513D, T1977R and N2043S) were located at functionally and structurally important regions of the protein as their conservation scores were high. Likewise, from the UniProt database, it was found that three of the polymorphisms were located within regions that were vital for interacting with other proteins (Fig 5). N2043S lies within a region that interacts with FKBP1A/rapamycin complex while L509Q and R619C are located in a region that interacts with NBS1 protein which is essential for DNA double-strand break repair [64]. The polymorphisms may affect the protein’s structure at these particular sites and hence interaction with the other molecules. On the other hand, K1452N, A1513D, E1610K, and R1616C variations were found within a core domain, FAT (Fig 5), which suggests these alterations may affect the protein’s core structure and function. Furthermore, whether these amino acid positions were exposed on the protein’s surface or buried within the protein, the surface accessibility of the residues was checked. Some residues were found to be exposed, indicating their potential impact on interaction with other molecules. Others were found to be buried within the protein showing their possible roles in maintaining the core structure of the protein.

All of the nsSNPs were found to decrease the mTOR protein’s stability as revealed by the negative free energy change values calculated in I-Mutant 3.0 and MUpro servers. Seven variations including L509Q, R619C, Y1151C, R1161G, A1513D, R1616C, and N2043S were shown to be largely destabilizing suggesting they might affect the protein’s folded structure. The structural effect of the selected variations was also analyzed by running HOPE projects. It was observed that eight of the 11 nsSNPs went through either a change in their charge or loss of their charge due to the alterations and that could result in interrupted interaction with other molecules. In addition, it could be seen that nine of these high-risk amino acid variations impacted either hydrogen bonds or salt bridges, or both within the mTOR protein. To further strengthen the predictions in this study, the MutPred2 tool was also applied which scores the probable pathogenicity of the amino acid variations showing the affected molecular mechanisms behind their pathogenicity. It was found that three of the mutations resulted in altered PTM sites, another four of the mutations resulted in the loss of transmembrane property and another one affected the secondary structure. Overall it could be seen that the deleterious nsSNPs could potentially affect the mTOR protein’s native structure and thereby function.

For a more detailed structural insight into the effect of the polymorphisms, the mutated models of the protein for the corresponding amino acid substitutions were generated in the SWISS-Model workspace. The RMSD and TM align values were calculated to determine the extent of change in the 3D protein structures due to the mutations. The RMSD value was quite low (less than or around 0.1) for all the mutated models suggesting that the mutant protein structures did not deviate much from the wild-type protein structure. However, for six of the amino acid substitutions, the TM align values came between 0.5 and 1 which indicated dissimilarity between the wild-type and mutant structures. Afterwards, three of these mutant structures including R619C, A1513D, and T1977R were further analyzed as their RMSD values were also comparatively higher than the other variant structures. Through structural analysis it was found that although the number of hydrogen bonds did not change between wild type and mutated residues, the bond angles, C alpha distance as well as the length of the H bonds changed due to the amino acid alterations further confirming that the polymorphisms may disrupt the protein’s native structure and stability. Several studies have shown that the interplay between protein backbone geometry and local conformation has an important contribution to protein stability [65]. Since backbone geometry can be affected by bond distances, bond angles, and dihedral angles, changes in such parameters resulting from the selected amino acid substitutions may affect the mTOR protein’s backbone geometry. This in turn may affect the protein’s local conformation and thus stability. The protein models were also analyzed in the Missense 3D tool and structural damage was predicted for the same three mutations: R619C, A1513D, and T1977R. In summary it can be said that these three SNPs could potentially impact mTOR protein’s functions as they have been found structurally and functionally deleterious. It should be noted here that data from various cancer-related databases also showed that these three mutations have been found to be associated with multiple types of cancers including renal clear cell carcinoma, melanoma, uterine endometrioid carcinoma, acute lymphoid leukemia, lung adenocarcinoma, and colorectal carcinoma [66–68].

Among the non-coding SNPs, rs2295079 was predicted to have a regulatory role over mTOR protein as it had a prediction of transcription binding sites, matched or unmatched motifs, and DNase footprint with DNase peak. On the other hand, rs12139042 was predicted to either disrupt a conserved miRNA site or create a new miRNA site. By altering miRNA binding sites, the particular SNP may hamper the regulation of the mTOR protein by miRNA.

This study has several limitations. For instance, due to variable penetrance, some disease- causing alleles do not manifest into a detectable phenotype, and therefore phenotypes cannot be entirely predicted based on genotype. Moreover, a single nsSNP is unlikely to provide insight into the phenotype of complex genetic traits like diabetes, heart disease etc. In addition to that, a common mutation may have a variable expression and diverse effects in different organs and tissue types of the body [14]. Therefore, efficient experimental analysis needs to be carried out in the future to ascertain the disease-associated nsSNPs.

## 5. Conclusion

In this study, several different bioinformatics tools were combined to screen for the most deleterious SNPs located within the mTOR protein. In total, eleven nsSNPs were filtered out to be the most high-risk as they affected the native protein’s structure and thereby function. Among these mutations, R619C, A1513D and T1977R were shown to cause structural damage to the protein while in addition to these three, L509Q, Y1151C, R1161G, R1616C and N2043S were also found to confer destabilizing effect over the protein. Furthermore, due to their position within interacting regions of mTOR, mutations like L509Q, R619C and N2043S may affect mTOR protein’s interaction with NBS1 protein and FKBP1A/rapamycin complex. Previous studies have associated several of these mutations with different cancer types however, D944V and N2043S have not been linked with any cancer yet, although they have shown disrupting structural consequences over the protein. These SNPs along with non-coding SNPs predicted to have detrimental effects on the protein structure, need to be further validated through laboratory experiments to confirm their roles in the pathogenesis of related diseases. The findings can serve as an important guide for the development of potential diagnostic and therapeutic interventions targeting these functional SNPs. All in all, this study has several promising prospects. One prospect is- based on these findings a guideline can be prepared to detail all the deleterious SNPs within mTOR which can potentially increase the risk of cancers and other disease susceptibilities. Another prospect is to perform extensive population-based studies involving clinical investigations to characterize the phenotypic effects of these genetic variations on the human population. And lastly, it will also be interesting to investigate the homozygosity and heterozygosity dependent pathogenicity level of these disease-associated mutations.

## Supporting information

S1 Fig Protein interaction network of mTOR protein. (TIF)

S2 Fig Ramachandran plot provided by procheck for wild-type mTOR protein. (TIFF)

S3 Fig Ramachandran plot provided by procheck for L509Q mutation. (TIFF)

S4 Fig Ramachandran plot provided by procheck for R619C mutation. (TIFF)

S5 Fig Ramachandran plot provided by procheck for D944V mutation. (TIFF)

S6 Fig Ramachandran plot provided by procheck for Y1151C mutation. (TIFF)

S7 Fig Ramachandran plot provided by procheck for R1161G mutation. (TIFF)

S8 Fig Ramachandran plot provided by procheck for K1452N mutation. (TIFF)

S9 Fig Ramachandran plot provided by procheck for A1513D mutation. (TIFF)

S10 Fig Ramachandran plot provided by procheck for E1610K mutation. (TIFF)

S11 Fig Ramachandran plot provided by procheck for R1616C mutation. (TIFF)

S12 Fig Ramachandran plot provided by procheck forT1977R mutation. (TIFF)

S13 Fig Ramachandran plot provided by procheck for N2043S mutation. (TIFF)

S14 Fig Superimposed structures of R619C mutant and wild type mTOR protein. (TIF)

S15 Fig Superimposed structures of A1513D mutant and wild type mTOR protein. (TIF)

S16 Fig Superimposed structures of T1977R mutant and wild type mTOR protein. (TIF)

S1 Table SIFT and PolyPhen-2 filtered nsSNPs along with their dbSNP IDs and scores derived from eight different in silico bioinformatics tools. (DOCX)

S2 Table Results of MutPred2 analysis of the 11 nsSNPs including their MutPred2 score and their impact on different molecular mechanisms. (DOCX)
