# Supplementary figures and images for: In silico comprehensive analysis of coding and non-coding SNPs in human mTOR protein
Source: PLoS One. 2022 Jul 5;17(7):e0270919. doi: 10.1371/journal.pone.0270919 (PMC9255762; doi:10.1371/journal.pone.0270919)

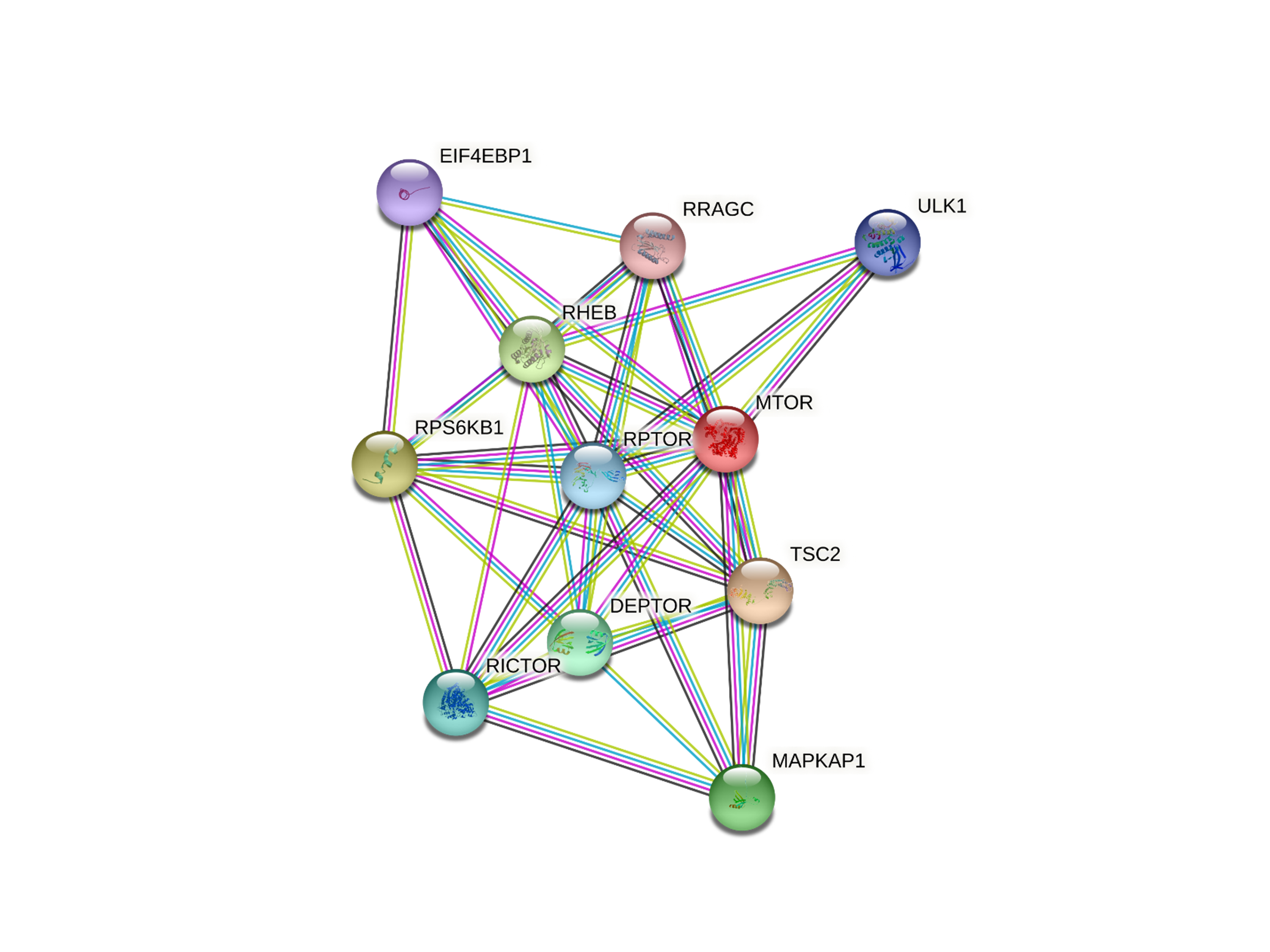

Supplement: S1 Fig — (TIF) [file pone.0270919.s001.tif]

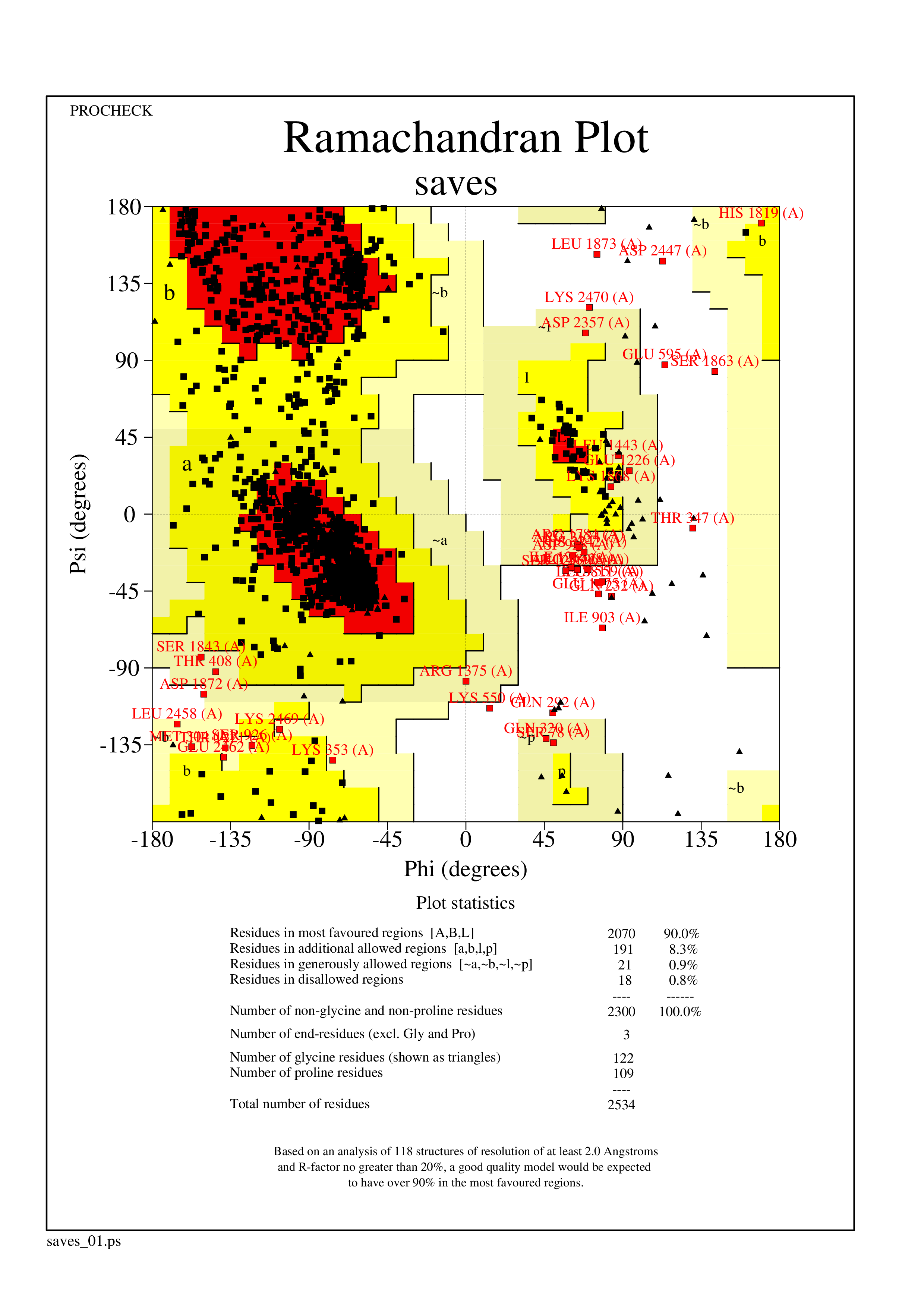

Supplement: S2 Fig — (TIFF) [file pone.0270919.s002.tiff]

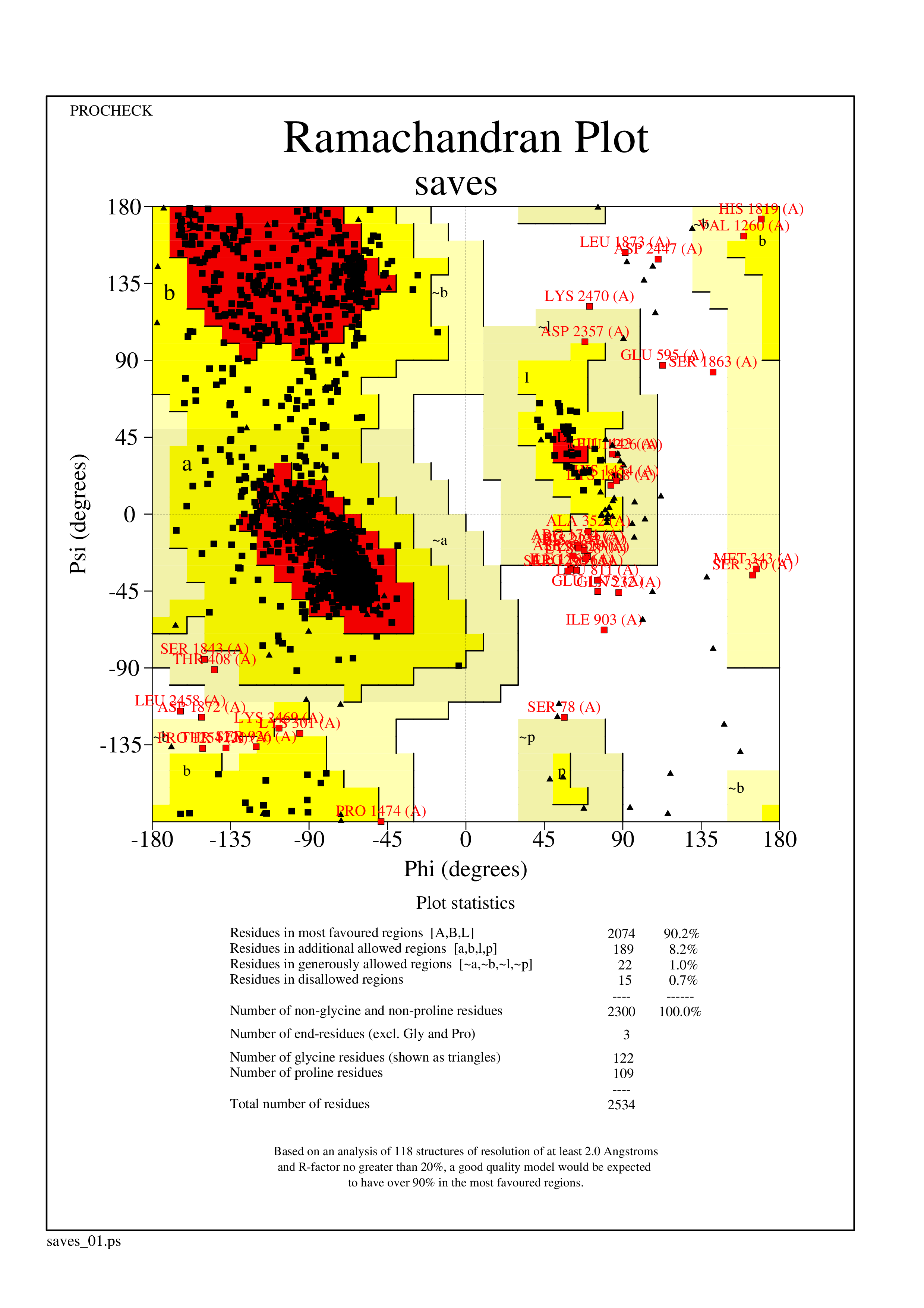

Supplement: S3 Fig — (TIFF) [file pone.0270919.s003.tiff]

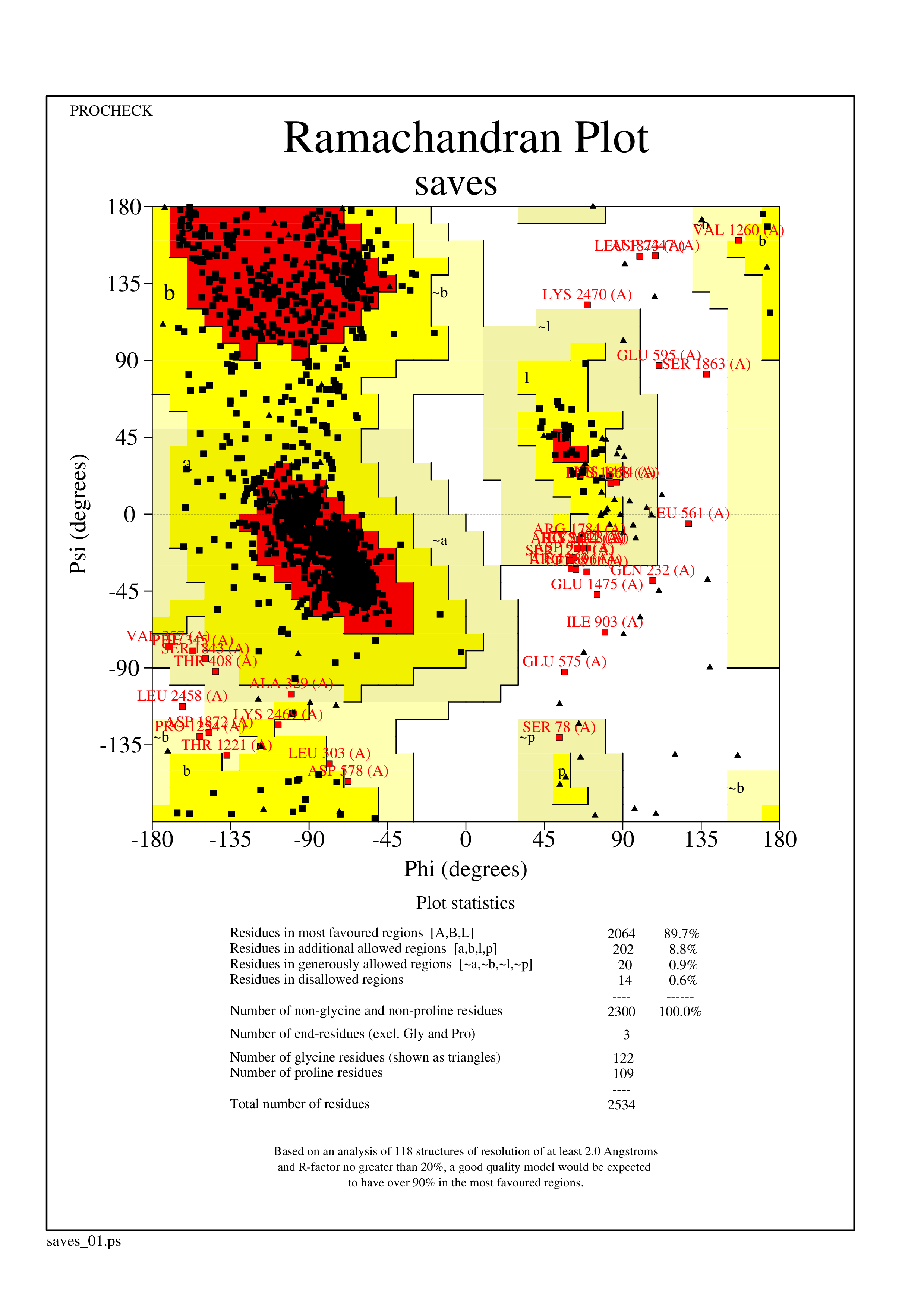

Supplement: S4 Fig — (TIFF) [file pone.0270919.s004.tiff]

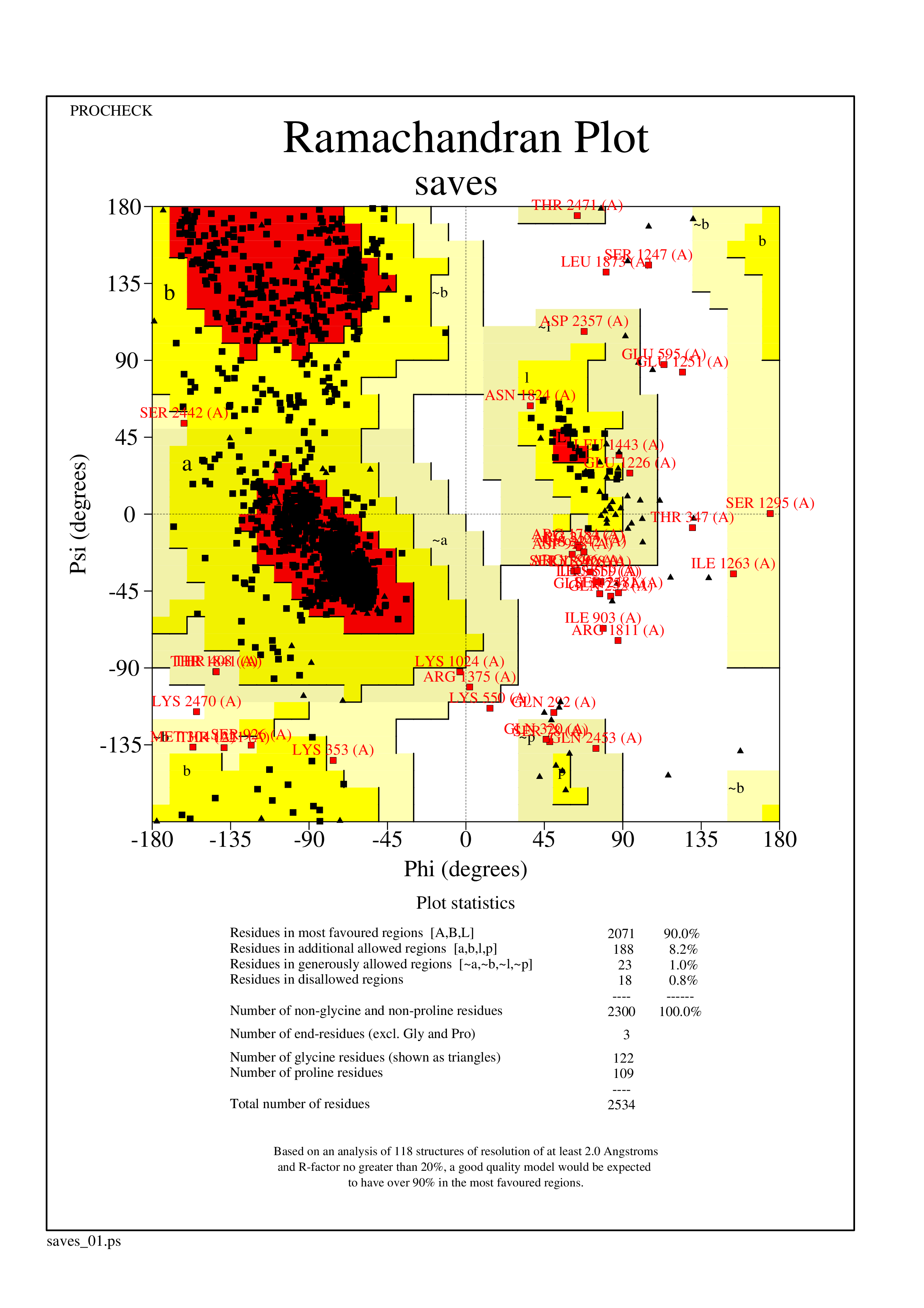

Supplement: S5 Fig — (TIFF) [file pone.0270919.s005.tiff]

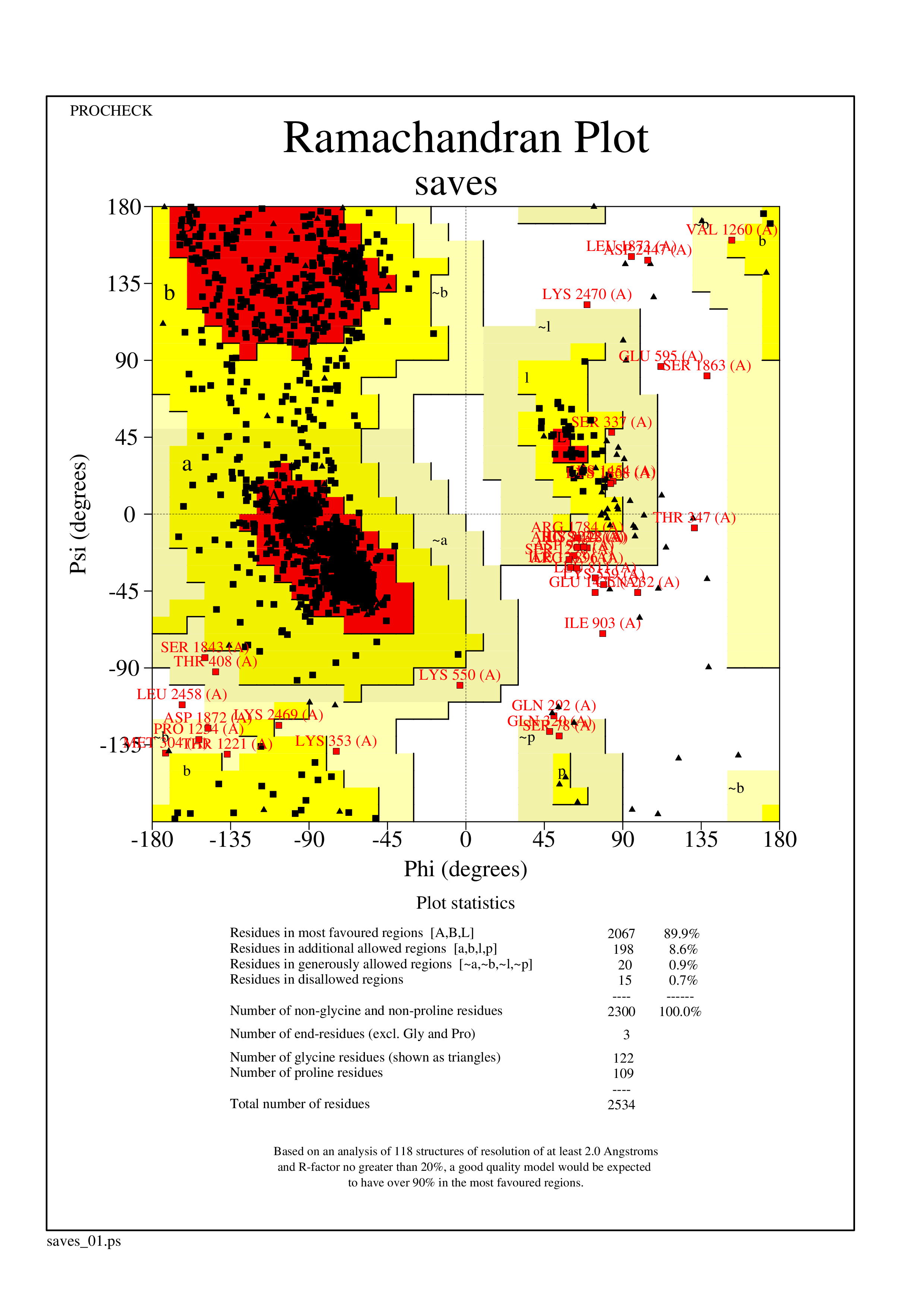

Supplement: S6 Fig — (TIFF) [file pone.0270919.s006.tiff]

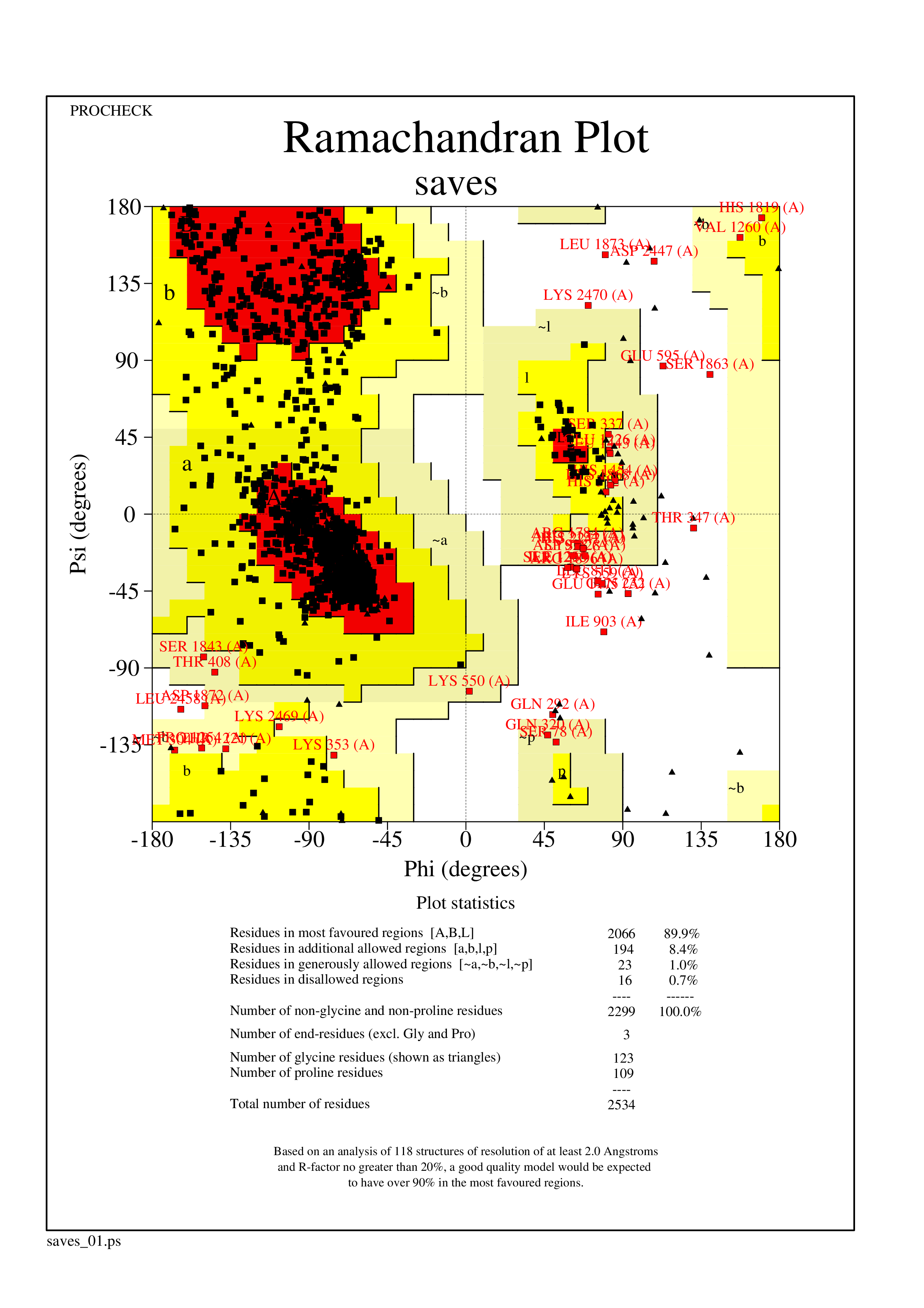

Supplement: S7 Fig — (TIFF) [file pone.0270919.s007.tiff]

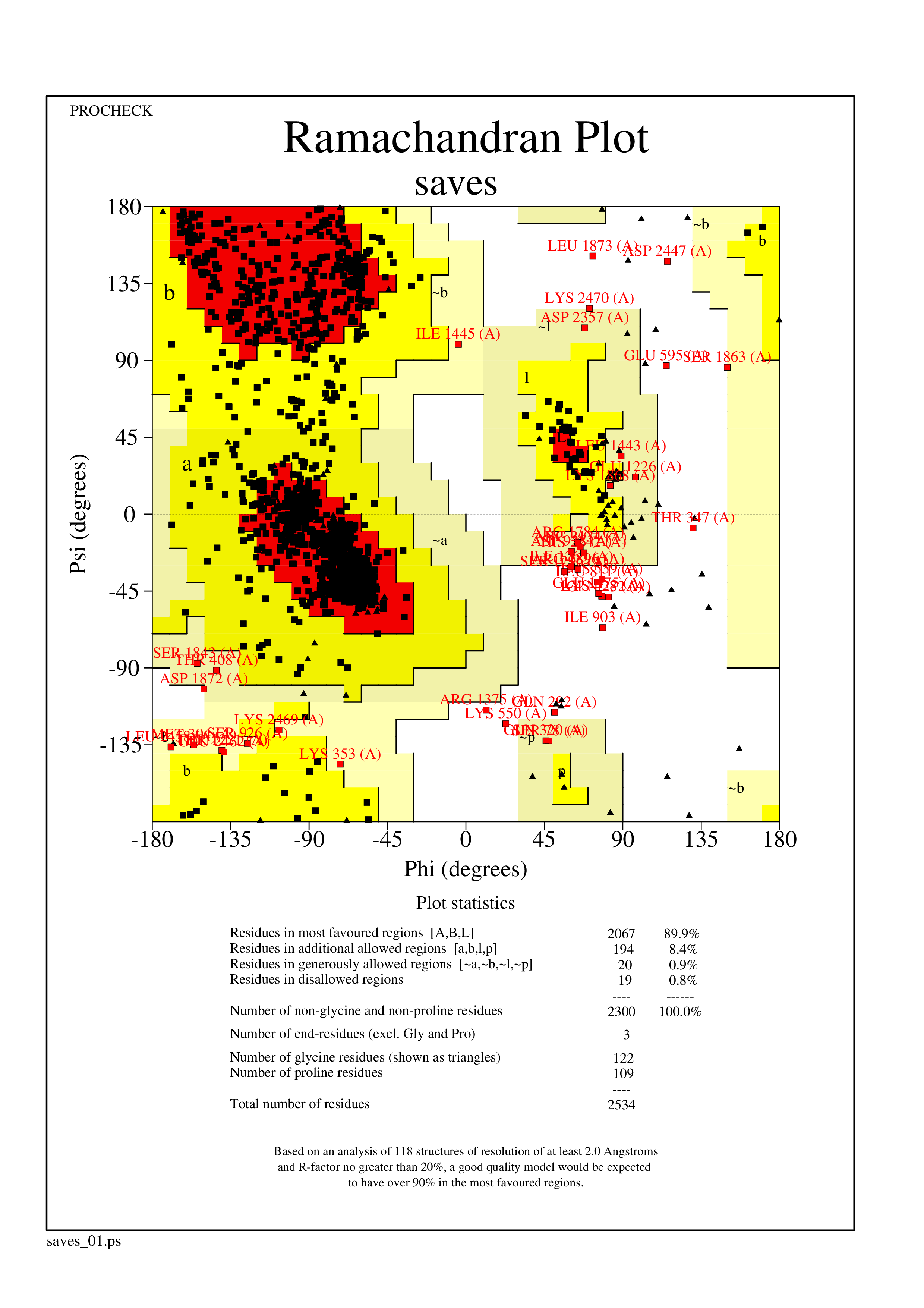

Supplement: S8 Fig — (TIFF) [file pone.0270919.s008.tiff]

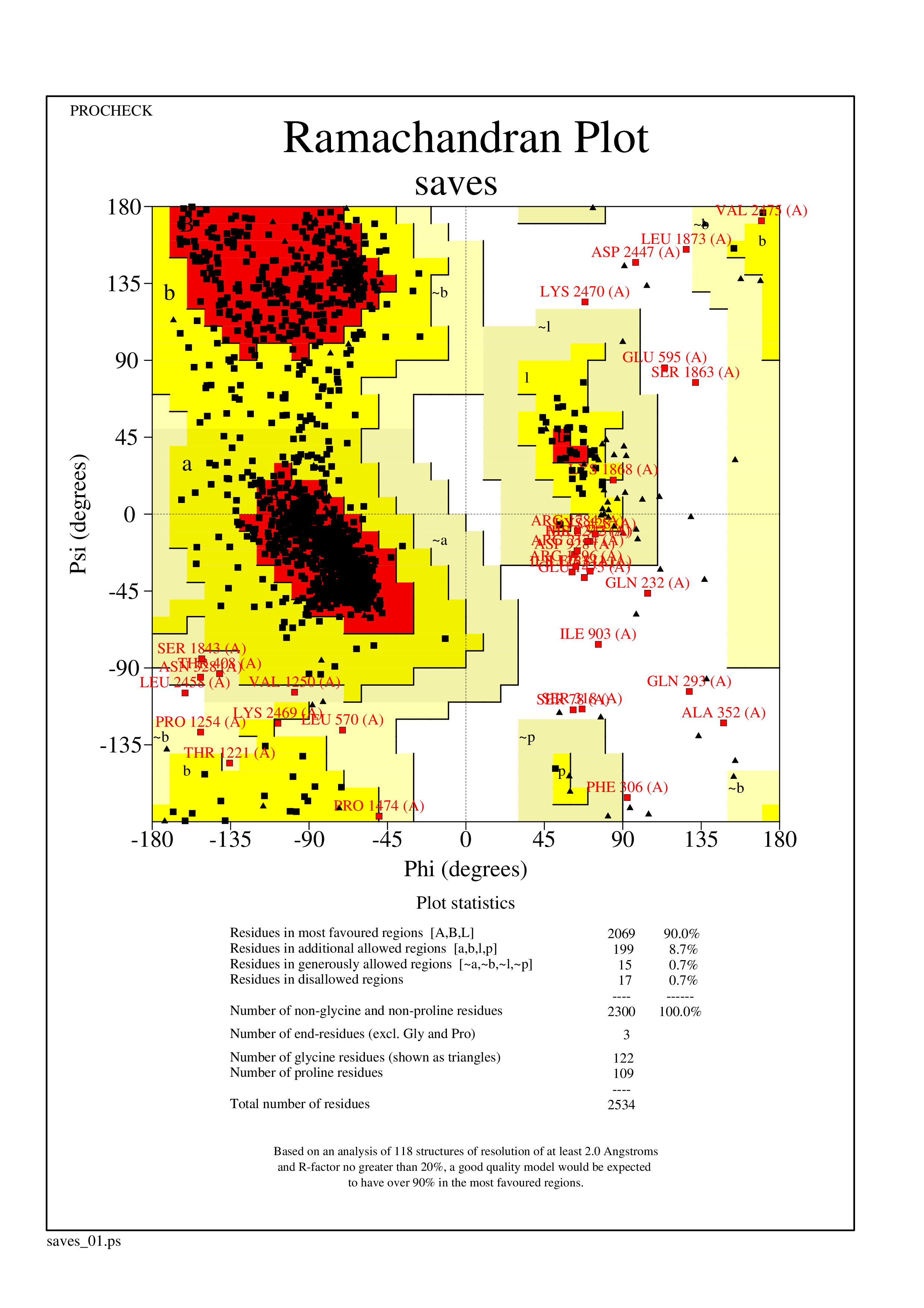

Supplement: S9 Fig — (TIFF) [file pone.0270919.s009.tiff]

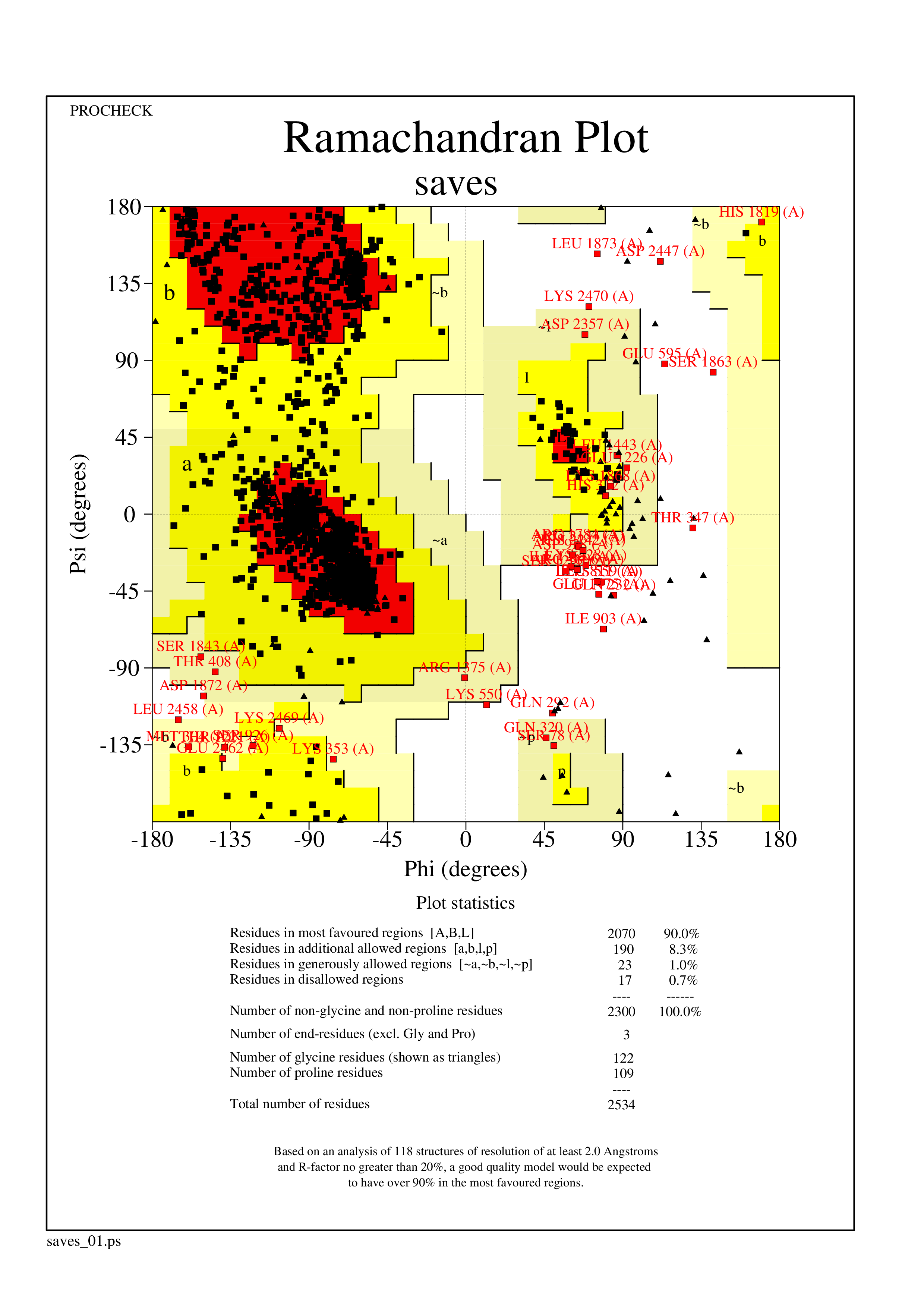

Supplement: S10 Fig — (TIFF) [file pone.0270919.s010.tiff]

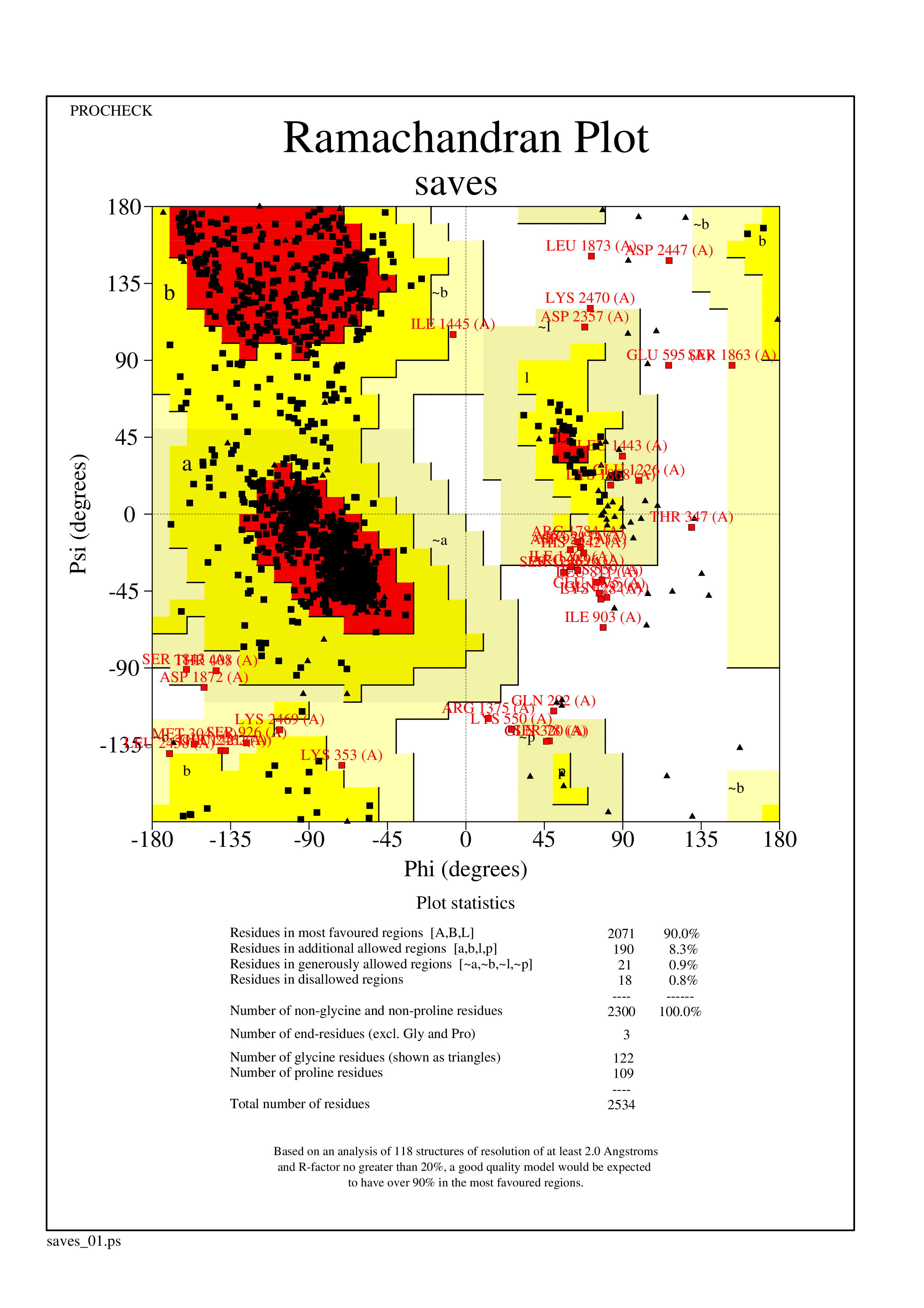

Supplement: S11 Fig — (TIFF) [file pone.0270919.s011.tiff]

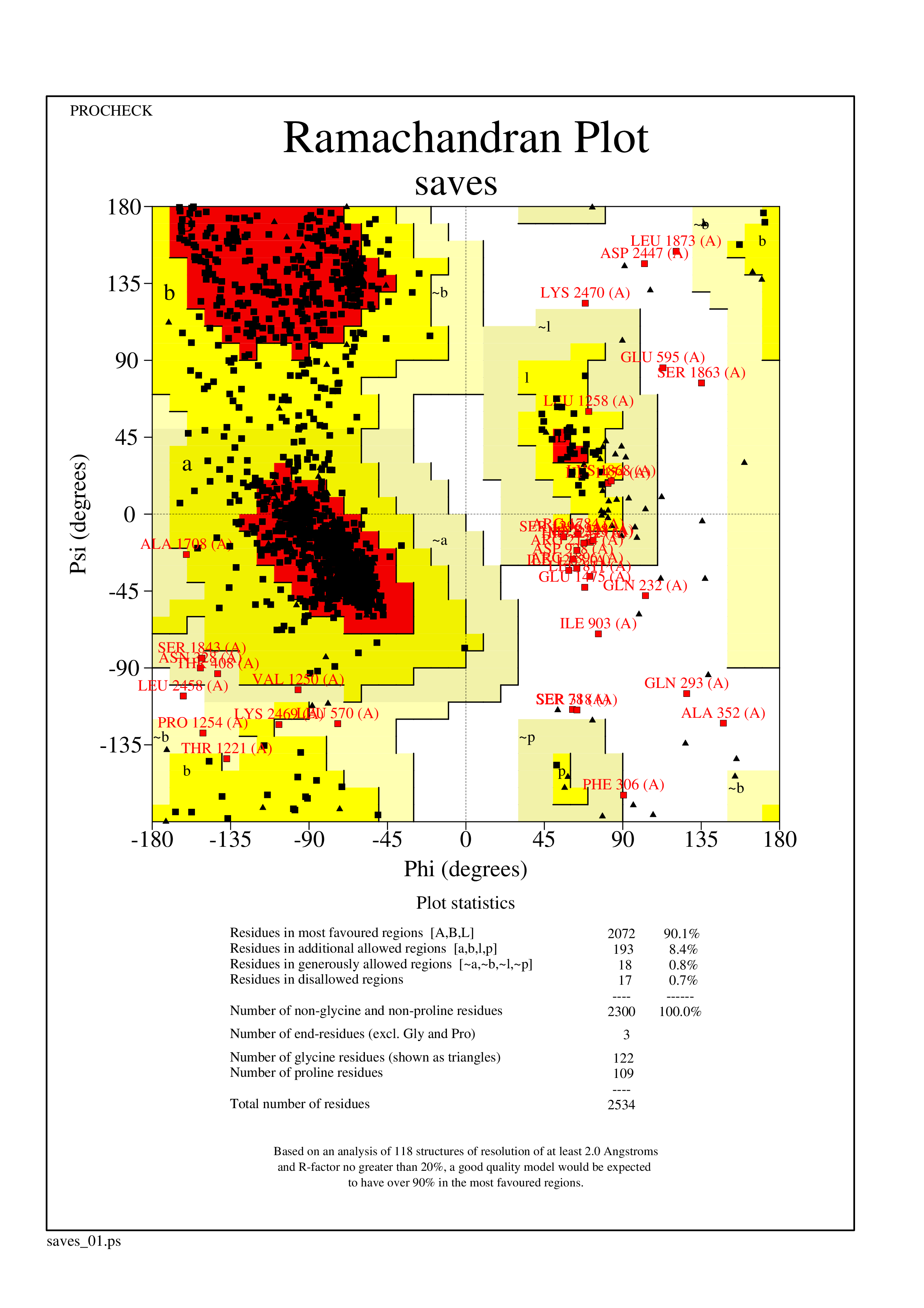

Supplement: S12 Fig — (TIFF) [file pone.0270919.s012.tiff]

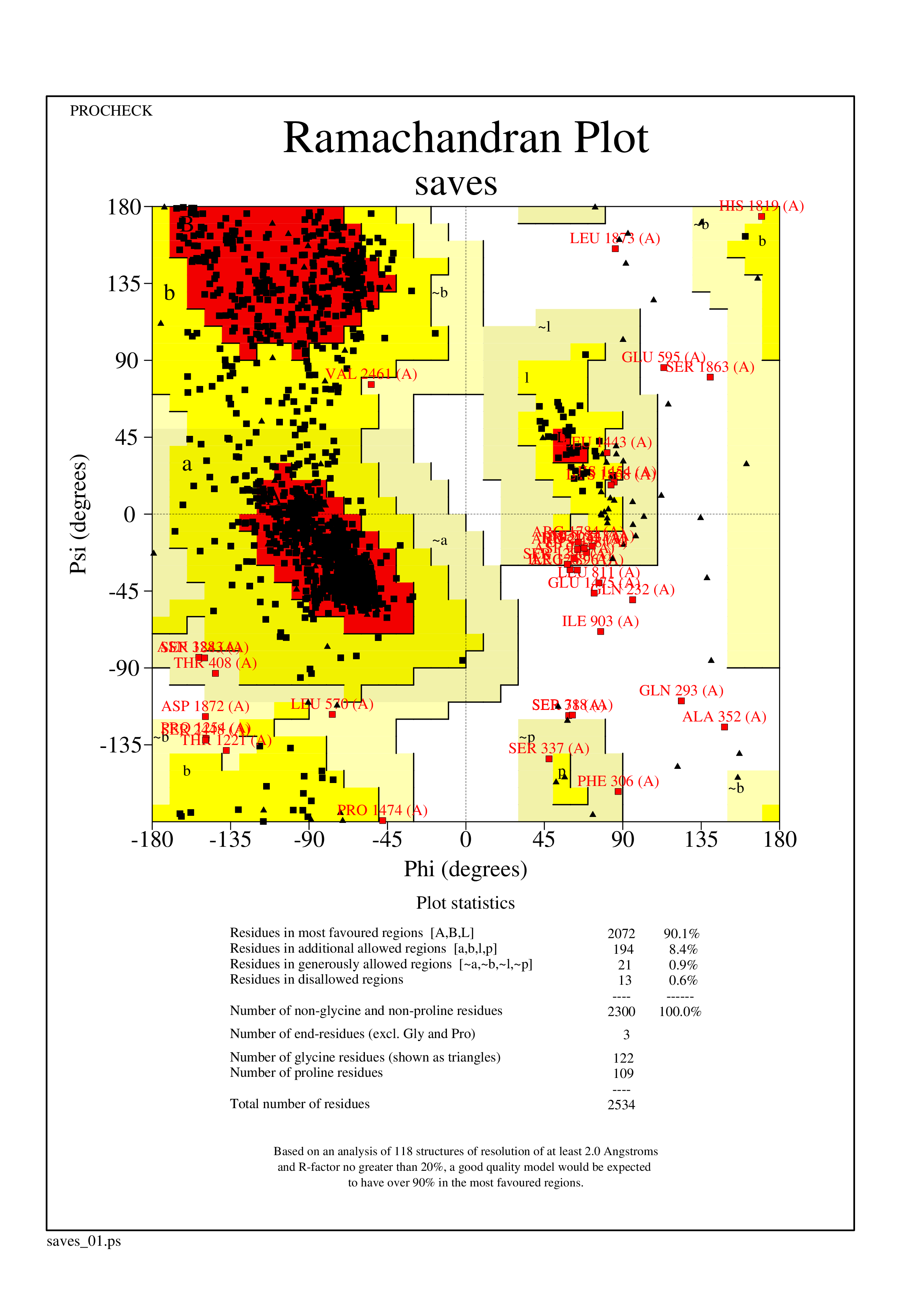

Supplement: S13 Fig — (TIFF) [file pone.0270919.s013.tiff]

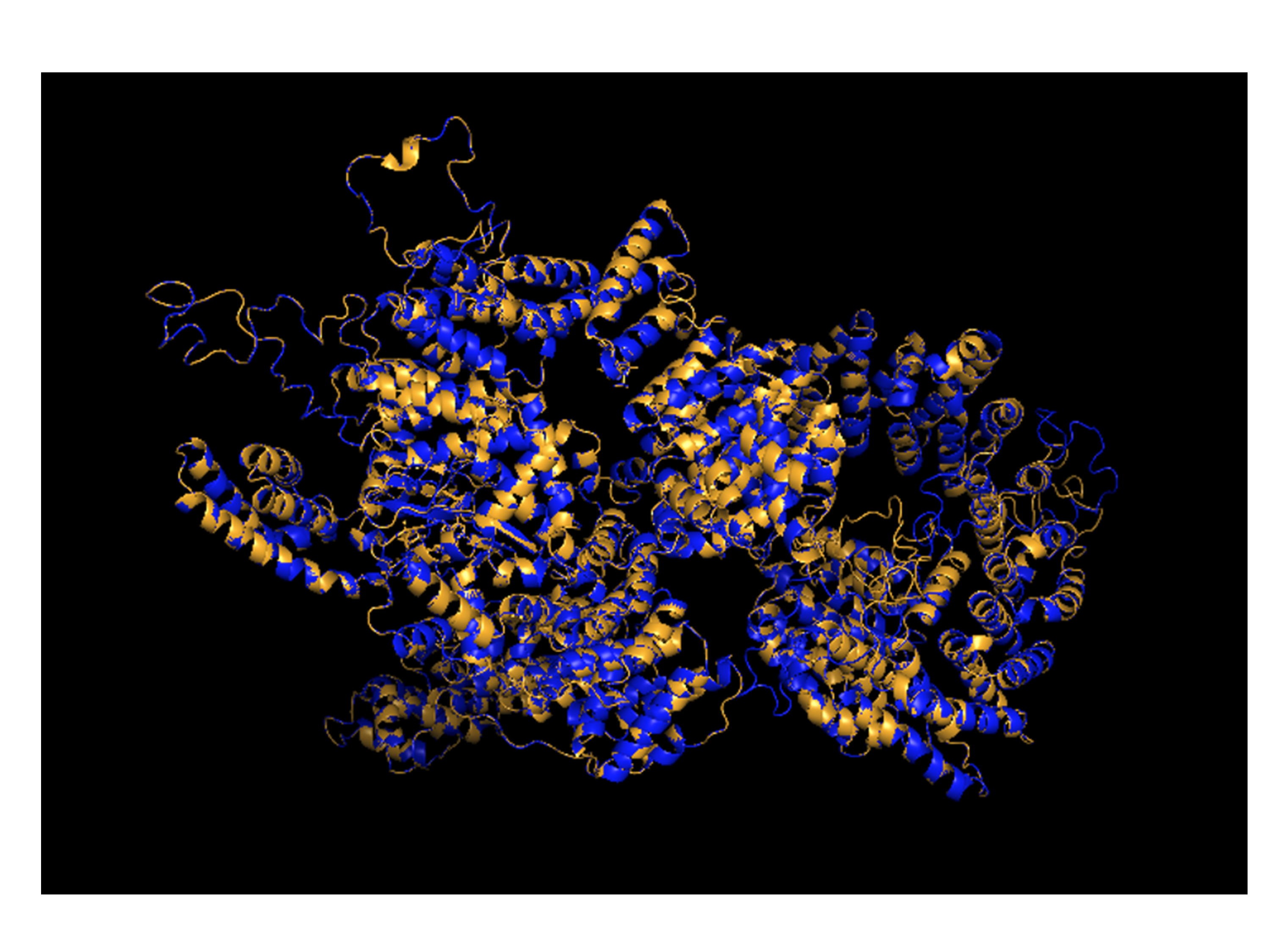

Supplement: S14 Fig — (TIF) [file pone.0270919.s014.tif]

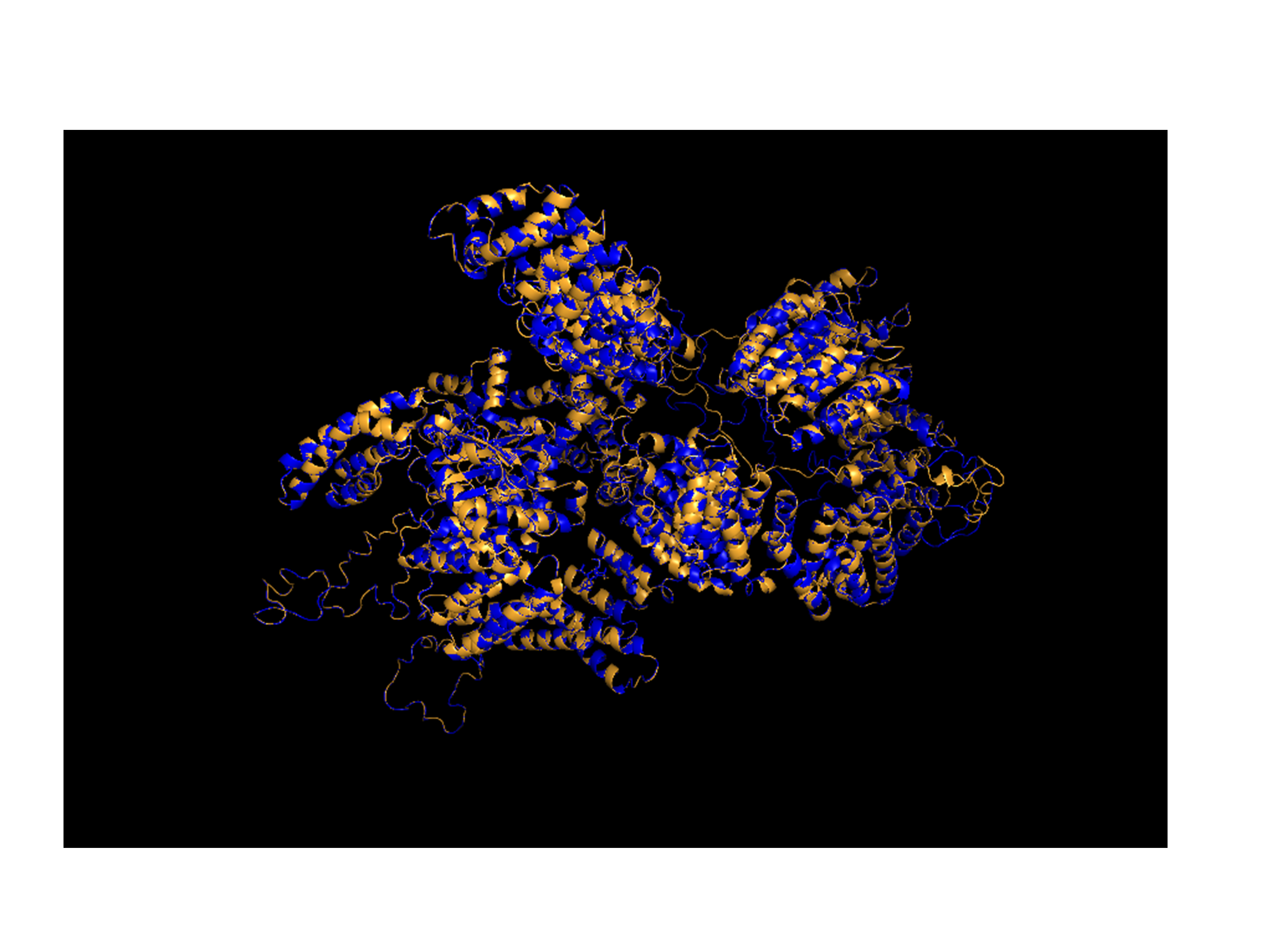

Supplement: S15 Fig — (TIF) [file pone.0270919.s015.tif]
