# Supplementary material for: In silico comprehensive analysis of coding and non-coding SNPs in human mTOR protein
Source: PLoS One. 2022 Jul 5;17(7):e0270919. doi: 10.1371/journal.pone.0270919 (PMC9255762; doi:10.1371/journal.pone.0270919)
Supplement: S1 Table — (DOCX) [file pone.0270919.s017.docx]

**S1 Table: SIFT and PolyPhen-2 filtered nsSNPs along with their dbSNP IDs and scores derived from eight different *in silico* bioinformatics tools**

| **AA variation** | **dbSNP ID** | **SIFT**  **score** | **PolyPhen2** | **PROVEAN** | **Mutation Assessor** | **SNAP2**  **(score)** | **SuSPect** | **PhD-SNP** | **PMut**  **(score)** | **CADD (PHRAD)** | **Meta-SNP (score)** |
| --- | --- | --- | --- | --- | --- | --- | --- | --- | --- | --- | --- |
| I2501F | - | 0.01 | 0.991 | -3.198 | medium | effect (45) | 65 | disease | disease (0.56) | 27.2 | neutral |
| R2316Q | 371628273 | 0 | 1 | -3.555 | medium | effect (56) | 85 | neutral | disease (0.73) | 33 | disease (0.682) |
| T2207S | 373990757 | 0.01 | 0.998 | -3.177 | medium | neutral | 43 | neutral | disease (0.61) | 26.6 | neutral |
| Q2194L | 113311044 | 0.03 | 0.557 | -5.979 | medium | effect (46) | 84 | neutral | disease (0.63) | 25 | neutral |
| P1694S | 373881875 | 0.022 | 0.997 | -6.677 | medium | neutral | 77 | disease | disease (0.73) | 29.5 | disease (0.525) |
| P1609R | 200634566 | 0.01 | 0.999 | -4.988 | medium | effect (1) | 44 | neutral | disease (0.54) | 29 | disease (0.565) |
| R1538W | 202187935 | 0.007 | 1 | -4.925 | medium | effect (55) | 42 | neutral | disease (0.79) | 32 | disease (0.713) |
| R1480C | 148486930 | 0.011 | 0.998 | -4.228 | medium | neutral | 70 | neutral | disease (0.54) | 26.1 | disease (0.595) |
| L1184V | 201856342 | 0.005 | 0.816 | -2.528 | medium | neutral | 83 | disease | disease (0.53) | 24 | disease (0.534) |
| S1178F | 55975118 | 0.003 | 0.952 | -2.619 | medium | effect (23) | 63 | disease | neutral | 25.7 | neutral |
| T1051M | 140743545 | 0.007 | 1 | -4.556 | medium | effect (14) | 63 | neutral | disease (0.69) | 28.4 | disease (0.708) |
| T996M | 376436307 | 0.028 | 0.999 | -3.132 | medium | effect (12) | 82 | neutral | disease (0.62) | 27.9 | neutral |
| Q992H | 368954451 | 0.016 | 0.998 | -4.371 | medium | effect (1) | 53 | disease | neutral | 23.3 | disease (0.554) |
| R886H | 376873727 | 0.003 | 1 | -4.626 | medium | effect (48) | 88 | neutral | neutral | 32 | disease (0.675) |
| K860M | - | 0 | 0.998 | -4.427 | medium | effect (11) | 67 | neutral | neutral | 28.5 | disease (0.556) |
| I802M | 142794621 | 0.003 | 0.98 | -2.575 | medium | neutral | 79 | neutral | disease (0.60) | 23.8 | disease (0.521) |
| S591R | - | 0.02 | 0.879 | -3.486 | low | effect (50) | 43 | disease | neutral | 23.7 | disease (0.523) |
| D523N | 376836258 | 0.033 | 0.961 | -3.343 | medium | effect (40) | 55 | neutral | neutral | 23.7 | neutral |
| R311H | 150146024 | 0.016 | 1 | -2.608 | low | neutral | 62 | disease | neutral | 31 | neutral |
| R311C | 200901835 | 0.002 | 1 | -3.972 | medium | neutral | 55 | disease | neutral | 32 | disease (0.655) |
| F182S | 369127640 | 0.008 | 0.992 | -5.358 | low | effect (2) | 49 | neutral | disease (0.57) | 23.7 | neutral |
| R165K | 144877536 | 0.046 | 0.992 | -2.62 | low | effect (78) | 74 | disease | neutral | 28.8 | disease  (0.55) |
| Y47C | 146812066 | 0.029 | 0.999 | -6.734 | medium | effect (12) | 81 | neutral | neutral | 27.7 | disease (0.715) |
| A1135G | - | 0.01 | 0.953 | -3.737 | medium | effect (50) | 75 | disease | neutral | 25.9 | disease (0.603) |
| R1784C | 371091288 | 0.036 | 0.913 | -3.024 | medium | effect (8) | 68 | neutral | disease (0.76) | 28.6 | disease (0.729) |
