# Supplementary material for: In silico comprehensive analysis of coding and non-coding SNPs in human mTOR protein
Source: PLoS One. 2022 Jul 5;17(7):e0270919. doi: 10.1371/journal.pone.0270919 (PMC9255762; doi:10.1371/journal.pone.0270919)
Supplement: S2 Table — (DOCX) [file pone.0270919.s018.docx]

**S2 Table: Results of MutPred2 analysis of the 11 nsSNPs including their MutPred2 score and their impact on different molecular mechanisms**

| **AA variation** | **MutPred2 score** | **Molecular mechanism with P values less than 0.05** |
| --- | --- | --- |
| L509Q | 0.938 | - |
| R619C | 0.950 | Loss of Acetylation at K616 |
| D944V | 0.861 | - |
| Y1151C | 0.865 | - |
| R1161G | 0.816 | - |
| K1452N | 0.748 | Altered Coiled coil  Altered Transmembrane protein |
| A1513D | 0.903 | Altered Transmembrane protein  Loss of Ubiquitination at K1511 |
| E1610K | 0.851 | Altered Disordered interface |
| R1616C | 0.858 | Altered Disordered interface  Altered Transmembrane protein |
| T1977R | 0.834 | Loss of Ubiquitination at K1981  Gain of ADP-ribosylation at T1977  Altered Transmembrane protein |
| N2043S | 0.725 | - |

MutPred2 score is the average of the scores from all neural networks in MutPred2. A score threshold of 0.50 would suggest pathogenicity.
